# Playing to the room: how solo pianists perceive, understand, and adapt to acoustic conditions

**DOI:** 10.3389/fpsyg.2026.1909431

**Published:** 2026-08-12

**Authors:** Mutong Shao, Aaron Williamon, George Waddell

**Affiliations:** Centre for Performance Science, Royal College of Music, London, United Kingdom

**Keywords:** acoustic simulation, acoustics, performance psychology, piano education, psychoacoustics

## Abstract

Musicians perform in vastly different acoustic environments. Therefore, the abilities to perceive and adapt to acoustic conditions are crucial to expert performance. Previous research has shown how room acoustics can influence performers' perception, preference, and performance behaviour, and that the sound heard by performers on stage may differ from the sound received by listeners elsewhere in the hall. This problem is intensified by daily preparation in practice spaces that may differ substantially from performance venues. This study investigated how solo pianists perceive acoustic conditions, interpret them through musical understanding, translate them into performance decisions, and develop skills of acoustical adaptation. Two linked qualitative phases were conducted. Phase 1 comprised semi-structured interviews with four conservatoire piano students and one piano professor. Phase 2 involved observations and reflective interviews with nine piano students across four contrasting acoustic scenarios. Acoustic simulation was achieved using the Royal College of Music (RCM) Performance Laboratory's venue-simulation technology, including a Meyer Sound Constellation electronically variable reverberation system, while instrument playback monitoring was conducted using audio, video, and a Steinway SPIRIO | r system. These scenarios served as situated prompts for interview-based reflection, while observation records, audio-video materials, and playback tasks contextualised participants' explanations of their adaptation strategies. Findings indicate that pianists' acoustical understanding was often tacit and musically grounded rather than closely aligned with standard acoustic terminology. Reverberation, decay, timbre, room support, and stage-audience discrepancy were understood mainly through their effects on tempo, pedalling, voicing, dynamics, projection, clarity, and communication. Adaptation strategies were shaped by practice environments, rehearsal access, room-instrument interaction, educational background, and audience-position feedback. The findings suggest that simulation technology, self-playing piano playback, structured room comparison, and targeted audience-position listening may support acoustical awareness by enabling comparison between different acoustic perspectives and linking reflective experience with developing performance knowledge. These results highlight the need for integrated research, teaching, and practice that links objective acoustic parameters with musical language and interpretation, performance decision-making, and pedagogical strategies.

## 1 Introduction

Musical performance and preparation occur across varied acoustic environments: practice rooms, teaching spaces, recording studios, concert halls, outdoor venues, and other unfamiliar settings. Research on room acoustics has defined and measured features including reverberation, clarity, support, intimacy, and early reflections (Ando, 1985; Beranek, 2004; International Organization for Standardization, 2009; Jordan, 1969; Meyer, 2009; Parkin, 1972; Sabine, 1906). Pianists interpret these features through playing: whether projection carries, whether resonance sustains a melody or reduces clarity between voices, and whether effort at the keyboard produces the intended sound at distance. This study examines how solo pianists interpret and respond to these consequences while playing, thereby providing a qualitative and phenomenological perspective on musician-acoustic interaction.

A particular challenge in understanding performers' relationship with their acoustic environment emerges from the disparity between performers' perception at the playing position on stage and listener perceptions across the venue, which are often prioritised in concert-space evaluation. Work in this area has distinguished performer and listener positions, examined acoustic support, reverberance, clarity, and mutual audibility, and shown why musicians' requirements cannot be inferred from audience-side evaluation alone (Dammerud, 2009; Gade, 1989a,b; Marshall et al., 1978; Ueno and Tachibana, 2003, 2005, 2011; Wenmaekers, 2017). Schärer Kalkandjiev and Weinzierl state the distinction clearly:

“musicians and audiences occupy different hall positions and have different involvement in the performance situation.” (2013, p. 1)

They also warn that musicians' primary response to room acoustics may appear in playing rather than verbal description, and that performer terminology can be diverse.

The issue is especially pronounced for solo pianists. Unlike many instrumentalists, pianists usually do not bring a familiar instrument to the venue. They must adapt to the room and to the venue piano at the same time. The feel of the key touch and action, timbre (i.e., nature of the tone), pedal response, balance across the range of pitches, and sound projection may all differ from the conditions in which the repertoire was prepared. The repertoire can make this adjustment especially demanding because inner-voice audibility, pitch colour, pedalling, and fine dynamic control often determine whether the musical texture remains clear. Research with singers and choirs also shows that performer-side acoustic demands vary by musical role. In choral singing, performers must negotiate the balance between their own voice and surrounding ensemble sound, with preferred self-to-other ratios varying between singers (Ternström, 1999). Classically trained singers have also been shown to alter aspects of vocal production across performance spaces, with room-related associations reported for vibrato extent and pitch inaccuracy alongside substantial individual differences (Bottalico et al., 2022). Pianists present a distinct case because they adapt through a venue-provided instrument while judging sound from a listening position different from that of the audience. These distinctions indicate that acoustical adaptation should not be assumed to operate uniformly across musician groups.

Piano-specific research remains relatively limited, although Bolzinger et al. (1994) studied seven professional pianists under controlled variable-acoustic conditions and found that room reverberance influenced playing intensity, while global tempo and contrast did not show significant variation. A qualitative question remains: how do pianists describe and make sense of their own adjustments when changes in room and instrument affect how their playing is perceived?

The educational literature on piano performance also recognises these concerns in teaching and practice. Neuhaus (1973) discussed the piano as capable of recreating the orchestra by differentiating tonal colour across pitches, rather than as a single uniform sound. This differentiation depends partly on whether the room allows contrasts of colour, line, and balance to remain audible. Schmitt (1893) gives a historical example of pedal pedagogy grounded in acoustical explanation, linking damper use to overtones, sympathetic vibration, tone duration, instrument condition, and the different effects of pedal use in large halls and small rooms. Banowetz (1985) later presents pedalling as a flexible decision shaped by the instrument, hall acoustics, and moment-by-moment response. These practitioner-oriented accounts frame room response as a musical problem: a room may affect loudness, reverberation, and the practical viability of colour, line, pitch balance, articulation, and pedal timing.

Another challenge in understanding musicians' relationship with acoustics is the practical and logistical difficulty of moving between the distinctive, often geographically dispersed spaces required to generate the acoustic stimulus. The Royal College of Music (RCM) Performance Laboratory and its associated venue simulations provide a practical setting for examining these issues. The RCM Performance Laboratory's simulation technology uses physical acoustic treatment and a Meyer Sound Constellation system to recreate different venue acoustics (Waddell et al., 2025a). Such facilities make it possible to examine acoustical adaptation through performance, playback, and reflection, rather than relying only on retrospective descriptions of venue experience.

Performer language raises a related pedagogical issue. Musicians may describe rooms as dry, wet, echoey, bright, supportive, dull, or blurred, but these terms do not always map neatly onto technical acoustic parameters. Such language organises acoustic knowledge through musical consequence: projection, balance between voices or across pitch, line clarity, and manageability under the hands. Schärer Kalkandjiev and Weinzierl (2013, p. 2) note that performer terminology can be diverse and that musicians' responses may appear in playing rather than verbal description. Ueno et al. (2010) linked musicians' comments on spatial impression, reverberation properties, sound propagation, and ease of performance to adjustments in tempo, articulation, note length, dynamics, and expression. Panton et al. (2019) examined chamber musicians' subjective impressions of auditorium stages in relation to reverberation time, early and late support, spatial energy distribution, and stage geometry. These studies support treating performer terms as practical labels for what the room makes playable, audible, or difficult. Pianist listening accounts support this attention to self-monitoring and auditory judgement (Sachs, 2009; Shao, 2020). The pedagogical question is how this practical knowledge can be made more explicit while keeping it tied to playing, rehearsing, and listening.

Acoustical adaptation is therefore understood as a form of practical musicianship. The focus is not hall preference alone, nor measurement of room response in isolation. This study examined how solo pianists understand acoustics, work under acoustic and practical constraints, adjust musical and technical parameters, and develop adaptive judgement through rehearsal, feedback, comparison, and technology-supported reflection. Using a two-phase qualitative design incorporating interviews and observations, it addressed the research question: How do solo pianists understand, respond to, and develop strategies for adapting to different acoustical environments?

## 2 Materials and methods

### 2.1 Research design

The study draws on tacit knowledge, reflective practice, ecological approaches to performance, and adaptive expertise to clarify the empirical problem. Polanyi's (1966) account of tacit knowledge helps explain why performers may find acoustical skill easier to enact than to verbalise: pianists use touch, resistance, returned sound, and pitch response while aiming towards a musical result, without isolating each cue separately. (Schön 1983) distinction between reflection-in-action and reflection-on-action provides a framework for understanding how performers monitor and evaluate acoustical conditions. In this study, moment-to-moment adjustments during playing, including changes to pedalling, voicing, tempo, touch, and projection, were considered forms of reflection-in-action. Post-scenario interviews, playback tasks, and audience-position listening provided opportunities for reflection-on-action by enabling participants to examine and reinterpret previous performance decisions. (Clarke 1988, 2005) ecological approach to musical performance positions the room and instrument as part of the performer's action environment, so perception and adjustment develop through repeated interaction with returned sound. (Hatano and Inagaki 1986) distinction between routine and adaptive expertise helps frame why previously successful performance strategies may require modification when room, piano, repertoire, or listener position changes.

A two-phase qualitative design was used. Phase 1 consisted of semi-structured interviews with four conservatoire piano students and one piano professor. Phase 2 consisted of semi-structured observations and reflective interviews with nine piano students working in contrasted acoustic settings. The two phases addressed the same performer-centred problem from complementary angles: what pianists say about acoustics, what they do when acoustic conditions change, and how they explain those actions afterwards.

### 2.2 Participants

Participants were recruited through the Royal College of Music (RCM). Phase 1 interviews were conducted remotely, while Phase 2 observations were conducted in the RCM Performance Laboratory.

Phase 1 involved four conservatoire piano students (S1–S4) and one piano professor (P1). The student participants comprised one Bachelor of Music student, two doctoral students, and one Artist Diploma student. Two participants were female and three were male. Phase 2 involved nine RCM piano students (S5–S13) who participated in observation and reflective-interview sessions under contrasted acoustic conditions. The Phase 2 student participants represented diverse international backgrounds, including five Chinese participants, one Australian participant, one UK participant, one Bulgarian participant, and one Lithuanian participant. Their training levels comprised four doctoral students, two Artist Diploma students, one Master of Performance student, and two Bachelor of Music students. Seven participants were female and two were male. Across both phases, participants represented different stages of advanced conservatoire piano training and a range of international backgrounds. All participants provided informed consent. Ethical approval was granted by the Royal College of Music Research Ethics Committee following the guidelines of the British Psychological Society. The professor participant was included purposively to provide an experienced pedagogical perspective on how acoustical awareness is developed and taught; this participant was not intended to constitute a comparison group or represent piano professors as a population.

In this report, student participants are referred to as S1–S13, and the piano professor is referred to as P1. Phase 1 involved S1-S4 and P1, while Phase 2 involved S5–S13.

### 2.3 Phase 1: semi-structured interviews

Phase 1 interviews addressed participants' understanding of acoustics, attention to acoustical features in practice and performance, reported adaptation strategies, and accounts of how adaptive skill is learned. The interviews were semi-structured: all participants were asked about the same broad topic areas, while follow-up and probing questions were used to clarify participants' meanings, invite comparison between spaces, and ask participants to explain how they approached acoustical preparation and adaptation in their own practice.

The interviews formed the qualitative base for identifying participant terminology, practical concerns, and reported adaptation strategies.

The full indicative interview schedule is provided in Supplementary material 2. The first author conducted all Phase 1 interviews remotely via Microsoft Teams. Interviews lasted approximately 30–60 min and were audio-video recorded with participant consent, then transcribed for analysis. The same indicative schedule was used across participants, with follow-up questions adapted to each participant's experience. The same researcher led the Phase 2 scenarios and reflective interviews.

### 2.4 Phase 2: observations and reflective interviews

Phase 2 used semi-structured observations and reflective interviews across four contrasted acoustical scenario types. The observations were conducted in the RCM Performance Laboratory using its acoustic simulation technology, which combines acoustic treatment with a Meyer Sound Constellation system to recreate different venue acoustics, together with a Steinway SPIRIO | r system that enabled performance capture and self-playing playback tasks (Waddell et al., 2025a).

The observation phase included four scenario types: (1) practice in contrasting acoustic spaces; (2) performance in simulated acoustic conditions that changed during playing; (3) recording followed by playback from different listening positions; and (4) movement from practice-room preparation to performance in a hall. Reflective interviews before and after each scenario asked participants to describe their initial impressions of the acoustics, discuss possible adaptation strategies, and reflect afterwards on what they noticed and how they responded. The detailed observation scenarios and reflective-interview procedure are provided in Supplementary material 2.

Participants generally worked with repertoire they were already preparing, using a score or performing from memory according to their usual practice; some scenarios also allowed sight-reading where appropriate. These choices formed part of the immediate performance context rather than separate conditions for comparison. Procedural consistency was provided by the defined purpose and sequence of each scenario, the acoustic contrasts introduced, and the common pre- and post-scenario reflective prompts. Score use and repertoire familiarity were documented as contextual features and considered when interpreting how participants noticed, judged, and responded to acoustic conditions.

The indicative procedure allocated approximately 15–30 min to each observation scenario and approximately 5–15 min to each pre- and post-scenario reflective interview. Participants could take part in different combinations of scenarios, so total session duration varied.

### 2.5 Data collection and analysis

Data were collected through interview recordings, observation recordings, field notes, and reflective interviews. In scenarios that included playback or audience-position listening, selected performances captured and replayed through the Steinway SPIRIO | r system were reviewed with participants during reflective interviews to prompt comments on what they noticed from outside the keyboard position. Audio-video recordings and SPIRIO | r performance recordings were also reviewed during analysis to contextualise observation notes and participants' reflective accounts. Field notes documented contextual information such as room acoustic simulation settings, task order, environmental constraints, and comments made between tasks or after playback.

Interview recordings were transcribed and anonymised before analysis. Chinese-language quotations were translated into English for presentation in this article. Direct quotations in the Results are identified by quote IDs that correspond to Supplementary material 1, which provides full transcription extracts, translations, and original-language wording where relevant.

Data from both phases were analysed thematically using Reflexive Thematic Analysis (Braun and Clarke, 2022). Analysis was conducted under the top-down framework of four interconnected domains driven by the research question and interview schedule design. These domains were (1) understanding of acoustics, (2) acoustic-related practise and performance constraints, (3) strategies for acoustical adaptation, and (4) the learning of acoustical awareness and adaptation processes. A bottom-up process of coding, sub-theme, and thematic generation was conducted within each of these domains.

The analysis followed a two-stage, iterative process. Phase 1 interview transcripts were first read and coded to identify how participants described acoustical perception, practice and performance constraints, adaptation strategies, and the development of adaptive skill. This analysis generated an initial coding and thematic framework, while coding remained open to participant terms, musical examples, and situational detail.

Phase 2 analysis followed the initial development of the Phase 1 coding framework. Observation records, reflective interviews, and associated performance materials were examined alongside the Phase 1 themes to connect participants' reported acoustical judgements with situated performance actions and subsequent reflection. This cross-phase analysis refined and extended the thematic structure by adding evidence of acoustic comparison, adaptation strategies, feedback routes, and reflective learning processes.

Reflexivity is a core requirement of RTA (Braun and Clarke, 2019). All coding and theme development for both phases were conducted by the first author; the study did not use independent dual coding, inter-rater reliability, or a consensus codebook. This is consistent with reflexive thematic analysis, in which coding is treated as an interpretive and iterative process rather than as a test of coder agreement. The first and third authors' experience as pianists supported recognition of performer-specific terminology and performance-practice context. Trustworthiness was supported through repeated engagement with transcripts and observation records, comparison of excerpts with their surrounding context, iterative versioning of the thematic structure and traceable linkage of cited quotations to Supplementary material 1.

## 3 Results

Table 1 summarises the four domains and emergent themes, while Tables 2–5 provide domain-specific summaries of themes and sub-themes. This article presents reduced excerpts from selected participant quotations together with quote IDs; full transcription extracts, translations, and relevant original-language contents are provided in Supplementary material 1. In each parenthetical marker, the participant code identifies the speaker, and the supplementary quote ID identifies the corresponding entry in Supplementary material 1; for example, (S1, SM1-S1-Q1) refers to Quote 1 from student participant S1. S1–S4 and P1 are Phase 1 sources, and S5–S13 are Phase 2 sources.

**Table 1:** Results domains and theme overview.

Domain	Themes
3.1 Domain 1. Solo pianists' understanding of acoustics	3.1.1 Listening and sensing acoustical feedback; 3.1.2 Discrepancies in understanding acoustics; 3.1.3 Understanding piano timbre and orchestral analogy; 3.1.4 Visual and inferred understanding of acoustics
3.2 Domain 2. Practising and performing under real-world constraints	3.2.1 Practice environments and habit formation; 3.2.2 Practical limitations of performance venues; 3.2.3 Discrepancies in acoustical perception; 3.2.4 Preference and performance effects under acoustical conditions; 3.2.5 Psychological effect of acoustics
3.3 Domain 3. Strategies and tools for acoustical adaptation	3.3.1 Pre-performance evaluation, rehearsal, and listening adjustment; 3.3.2 Principles and hierarchy within acoustical adaptation; 3.3.3 Adapting musical elements to ensure communication; 3.3.4 Instrumental and spatial adjustments; 3.3.5 Reflective listening, playback, and audience recalibration
3.4 Domain 4. Learning and developing acoustical awareness	3.4.1 Conservatoire training and the development of acoustical adaptation; 3.4.2 External references, comparison, and the growth of acoustical judgement; 3.4.3 Development of predictive judgement and self-monitoring; 3.4.4 Technology and self-evaluation tools; 3.4.5 Adaptation as a learnable skill

**Table 2:** Domain 1. Solo pianists' understanding of acoustics: themes and sub-themes.

Theme	Sub-themes
3.1.1 Listening and sensing acoustical feedback	3.1.1.1 Actively listening to acoustical response; 3.1.1.2 Physical sensation with instrumental feedback; 3.1.1.3 Individual part perception; 3.1.1.4 Dynamic response across the keyboard; 3.1.1.5 Projection of quiet sound
3.1.2 Discrepancies in understanding acoustics	3.1.2.1 Difference in terminology for describing acoustical phenomena; 3.1.2.2 Different approaches for perceiving acoustics; 3.1.2.3 Tacit knowing and partial verbalisation
3.1.3 Understanding piano timbre and orchestral analogy	3.1.3.1 Acoustical effect on the perception of instrumental timbre; 3.1.3.2 Orchestral thinking for piano timbre; 3.1.3.3 Piano's limitation in mimicking orchestral timbre; 3.1.3.4 Characteristics of different timbres of piano pitch
3.1.4 Visual and inferred understanding of acoustics	3.1.4.1 Visual indication of acoustical characteristics; 3.1.4.2 Architectural assumptions; 3.1.4.3 Audiences' hearing perspectives and stage-audience discrepancy

### 3.1 Domain 1. Solo pianists' understanding of acoustics

Participants described acoustic conditions through the musical judgements they made while playing: how sound returned from the room, how the instrument felt under the hands, how timbre changed, and whether projection and clarity seemed secure. Reflected sound, the dependability of the instrument, the clarity of individual voices, pitch behaviour, quiet projection, and the likely difference between keyboard-side and audience-side hearing all shaped how they understood the room. Acoustics entered their playing as a practical condition of interpretation rather than as a separate technical topic.

Table 2 summarises the themes and sub-themes within Domain 1.

#### 3.1.1 Theme: listening and sensing acoustical feedback

Participants primarily described room response through practical musical concerns: returned sound, touch at the keyboard, clarity of individual lines, decay across the keyboard, and whether quiet playing would carry. These concerns did not form stages in an adaptation sequence. They overlapped whenever performers judged whether the sound intended at the keyboard was produced in the room. The same concerns return in Section 3.3.1, Pre-performance evaluation, rehearsal, and listening adjustment, where pianists reported testing vulnerable passages before performance, and in Section 3.3.3, Adapting musical elements to ensure communication, where tempo, pedalling, voicing, dynamics, and projection become ways of managing room response.

##### 3.1.1.1 Actively listening to acoustical response

Participants reported judging acoustics through active room-listening. Acoustical feedback was something they sensed from the room and listened for while playing, especially when judging whether the room provided enough echo, reverberation, sustain, and clarity. The task involved deciding what that response would do to tempo, voicing, pedalling, and projection while playing.

“so I'm just trying to hear what's going on in the room if there is enough echo, if there is enough reverberation” (S1, SM1-S1-Q1)

##### 3.1.1.2 Physical sensation with instrumental feedback

Participants also described acoustical judgement through touch at the keyboard. The pianist cannot touch the room response directly while playing, so the instrument becomes the performer's immediate point of contact with the room's effect on sound. Key-depth, resistance, muscular effort, and the felt relation between movement and sound help the performer decide whether a difficulty belongs to the piano, the room, or the interaction between them.

“We can't touch the acoustics cause it's in the air [...] you need to be very, very comfortable with your hands and the instrument before you can start trying to adjust to acoustics” (S2, SM1-S2-Q1)

##### 3.1.1.3 Individual part perception

Participants connected acoustical clarity to whether internal musical relations remained audible. In polyphonic textures, the room was understood through the survival or loss of relationships between voices as well as through general resonance. Individual part-hearing offered pianists a musical reference for judging room response: they needed a clear internal model of the texture before they could notice how the room was changing it.

“how many people actually know what the alto voice sounds [like] against the bass voice [...] the tenor voice against the soprano? [...] it also shows how those voices interrelate.” (P1, SM1-P1-Q1)

##### 3.1.1.4 Dynamic response across the keyboard

Dynamic response differed across pitch and dynamic level. P1 described listening to the decay of sounds at different levels and in different areas of the keyboard as a form of aural training (SM1-P1-Q2). This kind of listening helps the pianist judge whether bass, middle, and treble areas remain balanced in the room. This pitch-specific listening becomes important again in Section 3.3.3.2, Clarity and voicing. A pianist cannot balance musical lines only by deciding which voice is important; they also have to judge how each pitch carries and decays in the room.

##### 3.1.1.5 Projection of quiet sound

Quiet projection acted as a particularly sensitive test of acoustic support. S1 framed quiet sound as a question of whether sound would reach the end of the hall, while S5 described how concert-hall support and practice-room dryness changed the practical meaning of softness. In these accounts, projection concerned more than volume: it involved whether a centred soft sound could travel without being forced.

“if I play something very quiet would it carry out to the end of the hall” (S1, SM1-S1-Q2)

“it's like an actor that can project even like whispering in a big hall that you will still hear it.” (S5, SM1-S5-Q1)

#### 3.1.2 Theme: discrepancies in understanding acoustics

When pianists tried to name or communicate what they noticed, discrepancies often appeared between technical acoustical vocabulary and performance language. Such language was analysed as evidence of practical acoustical understanding rather than as deficient terminology. Participants often expressed acoustical understanding through musical, bodily, spatial, or practical terms, which has implications for pedagogy and interdisciplinary communication. S2, for example, described one concert-hall acoustic as “extremely swimmy” and linked it to anxiety and the need to slow sections of the repertoire (SM1-S2-Q7; see Section 3.2.5.1). In the Phase 2 source coded S5, some halls were described as “less forgiving of you not having a center” (SM1-S5-Q4), while a dull acoustic was contrasted with one in which “I could sing because you can hear and you can connect the sounds between the two” (SM1-S5-Q5). These expressions connected perceived room response with centred projection, continuity between sounds, psychological state, and practical performance decisions, illustrating how performers articulated acoustical understanding through musical and experiential terms.

##### 3.1.2.1 Difference in terminology for describing acoustical phenomena

Participants often used musical, bodily, or practical language rather than stable technical terminology. Their descriptions organised acoustical meaning around consequence: what sound did to a phrase, a pitch, a texture, or a performance decision.

“Sound is really, really hard to quantify... especially in music... because everyone expresses things differently... so it is hard to quantify what the numbers should be.” (S3, SM1-S3-Q1)

##### 3.1.2.2 Different approaches for perceiving acoustics

One Phase 2 participant's account illustrates that performers may approach acoustics through different forms of musical judgement: room response, touch, pitch balance, timbre, and outside-listener comparison. Across the dataset, these forms appeared in different combinations, which suggests that adaptation depends on overlapping ways of listening and judging rather than a single general listening skill (SM1-S5-Q3).

##### 3.1.2.3 Tacit knowing and partial verbalisation

Tacit acoustic knowledge was often easier to enact than to verbalise. One Phase 2 reflection illustrates this difficulty: a performer could describe what they would do in a room more readily than isolate the acoustical property that triggered the response. The pedagogical implication is that effective practical knowledge can remain difficult to transmit unless teachers and students develop shared ways of naming the relevant cues.

#### 3.1.3 Theme: understanding piano timbre and orchestral analogy

Acoustical understanding also involved piano timbre. Participants used pitch colour, orchestral imagination, and timbral differentiation to organise the piano sound in space. The concern with individual parts in Section 3.1.1.3, Individual part perception, also shaped how participants talked about colour or the nature of the tone of each pitch. Distinct timbre could help keep internal strands audible when room response made pitch relations harder to separate.

##### 3.1.3.1 Acoustical effect on the perception of instrumental timbre

The room affected how the piano's timbre was perceived. A piano could seem brighter, duller, fuller, thinner, or less controlled depending on how the room supported or obscured the sound. Timbre sits alongside projection as a practical acoustical concern. A room can alter how pianists judge colour and balance, even when the instrument itself has not changed (SM1-S6-Q1).

##### 3.1.3.2 Orchestral thinking for piano timbre

Participants sometimes used orchestral analogies to manage piano colour. The analogy made internal balance and pitch differentiation more concrete: the pianist could imagine layers or voices as if they belonged to different instrumental groups within a single keyboard texture. This offers a timbral response to the problem raised in Section 3.1.1.3, Individual part perception: when room response threatens clarity, colour as well as volume can help preserve separation between musical lines.

“The more we think like an orchestra the better [...] In other words, you are in a sense, an orchestra.” (P1, SM1-P1-Q3)

##### 3.1.3.3 Piano's limitation in mimicking orchestral timbre

At the same time, orchestral analogy had limits. The piano can suggest orchestral colour, but it cannot reproduce sustained instrumental timbre directly. Acoustic support and decay affect how far the analogy remains convincing. A dry or unforgiving room may make the piano's attack and decay more exposed, while a resonant room may obscure the distinctions that the analogy is meant to clarify (SM1-P1-Q4).

##### 3.1.3.4 Characteristics of different timbres of piano pitch

Pianists did not treat the keyboard as a single uniform sound source. Bass, middle, and treble areas could respond differently in the same room, which affected how pitch colour, balance, and texture were judged. S6 treats pitch colour as part of acoustical judgement when balancing hands, lines, and textures (SM1-S6-Q2). These pitch differences become important again in Section 3.3.3.2, Clarity and voicing, and Section 3.3.3.3, Dynamics and projection, because balance and projection depend on how bass, middle, and treble material travel in the room.

#### 3.1.4 Theme: visual and inferred understanding of acoustics

Visual and spatial impressions could suggest an expectation of how the room might respond before full sound testing. Size, surface, furnishing, and architectural appearance could suggest likely room behaviour, but stage and audience hearing could still contradict those expectations. Visual expectation can become misleading when it is not checked through playing and listening. The same problem becomes a performance constraint in Section 3.2.3, Discrepancies in acoustical perception: what the room seems to promise visually may not match what the performer or listener actually hears.

##### 3.1.4.1 Visual indication of acoustical characteristics

Visual features of a room shaped provisional acoustical expectation. Size, surface, furnishing, and architectural appearance could suggest likely dryness, resonance, or projection before full testing occurred. These cues could orient performers, especially when rehearsal time was limited, but they could not replace active listening (SM1-S8-Q13).

##### 3.1.4.2 Architectural assumptions

Visual impressions sometimes operated as architectural assumptions. They could help performers form an expectation, but actual room response might contradict what the room appeared to promise. The active listening described in Section 3.1.1.1, actively listening to acoustical response, is where those assumptions are tested against sound. Visual judgement may guide attention but playing and listening decide whether that judgement is musically usable (SM1-S3-Q2).

##### 3.1.4.3 Audiences' hearing perspectives and stage-audience discrepancy

Audience perspective required both direct feedback and inference. S7 described teachers or listeners sitting farther back in rehearsals and concert halls and then reporting how the piano sounded from that position. Their account specified a learned translation between bench-side sound and hall-side result, rather than a general claim about audience perspective.

“*Usually [...] in the rehearsals and in the concert halls, they would sit closer towards the back and then they would report how it sounds from there.” (S7, SM1-S7-Q1)*

This feedback helped the performer build a practical translation between what was heard at the keyboard and what reached the audience:

“*you learn how it sounds here and then how it translates into the back of the hall.” (S7, SM1-S7-Q2)*

Their acoustical adaptation was therefore partly inferential: the pianist had to act on incomplete information while learning how stage impressions translated into hall sound. The discrepancy shaped the need for external listening, playback, and technology-supported recalibration.

“what you hear and what they hear can be very, very different” (S2, SM1-S2-Q2)

### 3.2 Domain 2. Practising and performing under real-world constraints

Adaptation took place under constraints that began before the concert: habitual practice rooms, limited access to halls, unfamiliar instruments, different audience and stage perspectives, preference, and psychological pressure. These constraints shaped what pianists could test, hear, and modify.

Table 3 summarises the themes and sub-themes within Domain 2.

**Table 3:** Domain 2. Practising and performing under real-world constraints: themes and sub-themes.

Theme	Sub-themes
3.2.1 Practice environments and habit formation	3.2.1.1 Acoustical constraints of practice rooms; 3.2.1.2 Noise distractions in practice environments; 3.2.1.3 Visual constraints of practice rooms
3.2.2 Practical limitations of performance venues	3.2.2.1 Predetermined and unmodifiable venue acoustics; 3.2.2.2 Predetermined instruments in performance venues; 3.2.2.3 Insufficient rehearsal time at the performance venue
3.2.3 Discrepancies in acoustical perception	3.2.3.1 Stage hearing does not equal hall hearing; 3.2.3.2 Difficulties of practice-to-performance transfer under acoustical change; 3.2.3.3 Ensemble coordination across listening positions
3.2.4 Preference and performance effects under acoustical conditions	3.2.4.1 Musicians' acoustical preference; 3.2.4.2 Underplaying tendencies; 3.2.4.3 Acoustical restrictions of musical expression; 3.2.4.4 Acoustical maladaptation due to fixated thinking; 3.2.4.5 Acoustical maladaptation due to cognitive overload
3.2.5 Psychological effect of acoustics	3.2.5.1 Anxiety caused by wet acoustics; 3.2.5.2 Anxiety caused by unpredictable acoustics; 3.2.5.3 Anxiety in competitive contexts affecting acoustical adaptation; 3.2.5.4 Confidence affecting acoustical adaptation

#### 3.2.1 Theme: practice environments and habit formation

Practice environments shaped pianists' default expectations about effort, sound, touch, and listening. Conservatoire practice spaces can be small, dry, noisy, visually uncomfortable, and acoustically distinct from performance venues. These rooms can train useful discipline, especially when the pianist has to work carefully for clarity and projection. They can also form misleading expectations about pedal use, dynamic scale, and effort. These habits become difficult in Section 3.2.3.2, Difficulties of practice-to-performance transfer under acoustical change, because touch, pedal, and dynamic effort learned in one room may no longer produce the intended result in different environments.

##### 3.2.1.1 Acoustical constraints of practice rooms

Practice-room acoustics shaped a pianist's default sense of how effort should translate into sound. A pianist who repeatedly practises in one room type may normalise a particular relation between touch, volume, pedal, and projection. That relation can become misleading in a hall, where the same physical effort may produce a different sound. For students, this risk is pronounced because daily practice can train the body before the performer has tested whether the habit works elsewhere.

“your muscles will get used to playing at a certain volume” (S1, SM1-S1-Q3)

S5 similarly described practice pods as unusually dry rather than equivalent to ordinary practice-room conditions:

“those pods are very drying [...] my environment is different than hers.” (S5, SM1-S5-Q7)

##### 3.2.1.2 Noise distractions in practice environments

Noise in practice environments added another constraint. External sound could interrupt listening, reduce confidence in self-monitoring, or force the pianist to ignore parts of the acoustical environment. Acoustical awareness depends on hearing fine differences in return, decay, balance, and projection. Distracting environments make that awareness less available during everyday preparation (SM1-S2-Q3).

##### 3.2.1.3 Visual constraints of practice rooms

Visual conditions formed part of the practice-room constraint. Poor lighting, enclosed rooms, or visually uninviting spaces could affect whether performers wanted to remain in the room long enough for focused work and could shape first impressions before sustained listening. This links to visual and inferred understanding in Domain 1: performers may use visual cues to predict how a room will sound, but those expectations still need to be tested through playing and listening. In practice rooms, the visual condition of the space therefore affected concentration and the performer's readiness to engage with the room as an acoustic environment (SM1-S12-Q18).

#### 3.2.2 Theme: practical limitations of performance venues

The constraints became more immediate in performance venues. In practice rooms, performers might gradually learn a space, adjust routines, or move to another room when access allowed. In a venue, the pianist usually encountered a fixed combination of room, piano, rehearsal schedule, and available support. The piano could be unfamiliar, rehearsal time short, and opportunities for listener feedback limited. The performer could still adjust the performance, but only within what could be changed from the keyboard. Venue adaptation was therefore shaped by the limited aspects of the situation that performers could modify from the keyboard.

##### 3.2.2.1 Predetermined and unmodifiable venue acoustics

In the performance situations described by participants, venue acoustics were normally treated as predetermined. Variable-acoustic venues and simulation systems exist, including IRCAM's Espace de Projection, the Gulbenkian Great Hall's variable stage configuration, and the RCM Performance Laboratory's venue-simulation technology, but these are specialist installations rather than ordinary performance conditions. S9 described the practical constraint that performers cannot redesign the room; they have to respond musically to what is already there (SM1-S9-Q1). The same constraint appears in S1′s account, where control rests in shaping the response rather than changing the room. When the room could not be changed, responsibility shifted back onto performance decisions: touch, timing, pedal, balance, projection, and rehearsal strategy.

“acoustics are not under your control... but what is under your control is your response to the acoustics.” (S1, SM1-S1-Q4)

##### 3.2.2.2 Predetermined instruments in performance venues

For pianists, room adaptation is inseparable from instrument adaptation. Unlike many instrumentalists, pianists usually perform on the venue's instrument. The pianist-specific complication is that unfamiliar touch, tonal balance, pedal response, and pitch behaviour may all interact with the room's acoustic response and can create challenges in disentangling the effects of the instrument from the effects of the room.

“it is not just the acoustic, it's the piano too. This is not your own instrument. It's another instrument.” (P1, SM1-P1-Q5)

##### 3.2.2.3 Insufficient rehearsal time at the performance venue

Limited rehearsal time made adaptation more uncertain. Performers could sometimes form a general idea of the room, but full calibration of projection, balance, tempo, and pedal was not always possible before performance. Rehearsal therefore became a practical compromise: identify the most vulnerable areas, then carry a partly tested strategy into performance.

“because we didn't have any rehearsal in the concert hall before the performance day, we didn't know how it was gonna sound.” (S1, SM1-S1-Q5)

#### 3.2.3 Theme: discrepancies in acoustical perception

The gap between keyboard-side hearing and listener-side sound becomes a practical performance constraint in this theme. Pianists make decisions from the stage while knowing that their hearing there may differ from listener-position hearing elsewhere in the hall. The audience-position concern in Section 3.1.4.3, Audiences' hearing perspectives and stage-audience discrepancy, becomes a performance risk here: the performer has to act from the keyboard while knowing that the sound they are trying to communicate will be judged elsewhere in the hall.

##### 3.2.3.1 Stage hearing does not equal hall hearing

Participants reported that stage hearing did not reliably equal hall hearing. A performer might feel that bass, treble, or inner material was sufficient on stage, only to learn later that the hall balance was different. Audience-side feedback therefore checked the limits of the performer's own listening position.

“… But to you on stage it sounded enough [...] there's just [...] no definite way of finding out by yourself how it sounds from the audience.” (S2, SM1-S2-Q4)

##### 3.2.3.2 Difficulties of practice-to-performance transfer under acoustical change

Transfer from practice to performance became difficult when the same musical passage required a different relation between touch, effort, and room support. S5 contrasted the practical meaning of soft playing in a concert hall with the effort needed in a practice room:

“With the softer passages [...] in like a concert hall, you can play softer then in that acoustic because you know it will carry. Whereas in a practice room, you have to project.” (S5, SM1-S5-Q2)

The difficulty lay in carrying a practice-room habit into a space where sound behaved differently. A passage learned through greater projection in a dry room could require less force in a supportive hall, while a sound that seemed secure in practice might need recalibration when resonance, distance, and audience-side hearing changed.

##### 3.2.3.3 Ensemble coordination across listening positions

Shared performance examples extended the issue of position-dependent hearing beyond the solo pianist's own stage position. Participants described how resonance, distance, and mutual audibility could affect coordination between performers, especially when players did not hear the same balance or timing from their respective positions. In these cases, the acoustic condition created a discrepancy between what each performer could hear and what the ensemble required for synchronisation (SM1-S1-Q10; SM1-S1-Q11).

The relevance to solo piano lies in the listening-position problem also described in Section 3.2.3.1. One acoustic environment can produce different versions of the same musical event for different listeners or performers. For solo pianists, this discrepancy is concentrated into the relation between keyboard-side judgement and hall-side result: they must make decisions from the bench while the sounded result is assessed elsewhere in the room.

#### 3.2.4 Theme: preference and performance effects under acoustical conditions

Preference and adaptation were related but not identical. Pianists could prefer supportive resonance, clarity, or a particular balance of room response, but those preferences did not by themselves produce reliable adaptation. A room that felt comfortable at the keyboard could still encourage underplaying, excessive trust in stage feedback, or attachment to a familiar sound that did not communicate in the venue. This theme therefore concerns the performance effects of acoustical preference and miscalibration: how performers' playing could change, or fail to change, when acoustic conditions shaped what felt sufficient, expressive, or secure.

##### 3.2.4.1 Musicians' acoustical preference

S5 described a preference for acoustic conditions that supported particular musical priorities (SM1-S5-Q4). Such preferences were contextual rather than universal: a room that supported one repertoire, pitch area, or performer could expose problems for another. Preference became analytically important when it shaped what the pianist trusted at the keyboard, rather than when it simply named a favoured type of room.

##### 3.2.4.2 Underplaying tendencies

Underplaying appeared when stage sensation made the sound feel fuller or louder than it was likely to be in the hall. This tendency could arise from the mismatch between bodily comfort, stage feedback, and audience-side projection. Recognising underplaying therefore depended on the performer's ability to translate between what felt sufficient at the keyboard and what carried outward.

“I used to have a problem with underplaying [...] not playing full out.” (S2, SM1-S2-Q5)

##### 3.2.4.3 Acoustical restrictions of musical expression

Acoustics could restrict musical expression by making certain interpretative choices less communicable. A wet room could blur rapid or harmonically dense material; a dry room could make legato or quiet connection harder to sustain. Such restriction did not remove artistic freedom, but it changed the conditions under which an expressive idea could be made audible (SM1-S5-Q5).

This sub-theme concerns the nature of the restriction, while Section 3.3.3, Adapting musical elements to ensure communication discusses approaches pianists used to reshape musical elements in response to such conditions.

##### 3.2.4.4 Acoustical maladaptation due to fixated thinking

Maladaptation could occur when a familiar practice-room sound or instrument–room relation became an assumed norm for a new venue. A pianist could bring a sound-image or technical answer formed elsewhere and judge the present room against that earlier condition. This connects back to Section 3.2.1, where repeated work in one acoustic setting could normalise a particular relation between effort, volume, pedal, and projection. P1 framed adaptation against this portability of an ideal sound:

“you can't take that around from piano to piano, from hall to hall… you have to be able to adapt… …we can't simply think… I played this way, this is my ideal way, this is my ideal acoustic… and you can't transport that you have to be able to adapt. And that is going to be a matter of profound listening.” (P1, SM1-P1-Q8)

A familiar sound may guide preparation, but it can become prejudicial when it reduces renewed listening to the piano, room, and listener condition actually present. The same risk returns in Section 3.4.5.4, where adaptation is treated as a learnable but non-transferable skill. Experience may help performers adapt more quickly, but it does not turn one preferred sound or one successful solution into a rule for later halls and pianos.

##### 3.2.4.5 Acoustical maladaptation due to cognitive overload

Cognitive overload was another route to maladaptation. Under pressure, performers had to monitor the score, memory, touch, sound, audience, and room response at once. If too many variables demanded attention, adaptation could become reactive or inconsistent. Rehearsal routines in Section 3.3.1, Pre-performance evaluation, rehearsal, and listening adjustment, can reduce this burden by identifying in advance which passages, sounds, or parameters most need to be tested (SM1-S7-Q4).

#### 3.2.5 Theme: psychological effect of acoustics

Acoustics affected both sound and psychological state. Participants described anxiety, uncertainty, competition pressure, and confidence as part of acoustical adaptation. These psychological dimensions were not separate from musicianship. They shaped the performer's capacity to listen, take risks, adjust quickly, and trust decisions in the moment.

##### 3.2.5.1 Anxiety caused by wet acoustics

In wet acoustics, strong reverberation prolonged the sound and could blur notes or textures into what followed. Participants described this as anxiety-provoking when resonance made them unsure whether texture, articulation, or tempo remained intelligible. The anxiety came from uncertainty about the sounding result after it left the instrument. When the sound became “swimmy,” the performer had to decide whether to slow down, articulate more clearly, reduce pedal, or accept a less precise result.

“Because it was extremely swimmy... that was the main anxiety point for everybody.” (S2, SM1-S2-Q7)

##### 3.2.5.2 Anxiety caused by unpredictable acoustics

Unpredictable acoustics caused a different kind of pressure. When the room behaved in ways the pianist had not anticipated, adaptation had to happen without enough rehearsal information. Uncertainty weakened the performer's own monitoring and increased reliance on quick testing, external listening, or familiar fallback strategies (SM1-S8-Q1).

##### 3.2.5.3 Anxiety in competitive contexts affecting acoustical adaptation

Competition and assessment contexts intensified acoustical anxiety by adding comparison with other performers. Difficulty came from the room itself and from the possibility that another performer might adjust more effectively. That comparison could narrow attention and make flexible listening harder.

“what if somebody is adjusting to this better than I'm adjusting to it?” (S2, SM1-S2-Q8)

##### 3.2.5.4 Confidence affecting acoustical adaptation

Confidence affected adaptation because the performer had to act before fully knowing whether the adjustment worked. S9 described being able to react quickly while still recognising that the effectiveness of the reaction remained uncertain. This uncertainty is closely related to Section 3.1.4.3, Audiences' hearing perspectives and stage-audience discrepancy audience hearing: the pianist often has to commit to an adjustment before its listener-position result can be fully checked.

“I was able to react quite quickly. Whether or not my adjustments were effective is a different matter.” (S9, SM1-S9-Q2)

### 3.3 Domain 3. Strategies and tools for acoustical adaptation

Participants responded to acoustic conditions through interrelated actions: testing the room, identifying vulnerable material, preserving the musical interpretation where possible, adjusting musical and technical parameters, using external feedback, and sometimes modifying the physical setup. These strategies responded to the constraints described in Domain 2, especially limits on room choice, rehearsal access, stage-audience translation, and performance security. The need for adaptation arose as the sounding result varied across practice spaces, venues, pianos, rehearsal schedules, audience presence, and listening positions. Domain 3 thus focuses on how pianists tested, recalibrated, and communicated musical intentions under changing conditions.

Table 4 summarises the themes and sub-themes within Domain 3.

**Table 4:** Domain 3. Strategies and tools for acoustical adaptation: themes and sub-themes.

Theme	Sub-themes
3.3.1 Pre-performance evaluation, rehearsal, and listening adjustment	3.3.1.1 Evaluating reverberation through impulse sounds; 3.3.1.2 Rehearsal for identifying a general idea of the adaptation; 3.3.1.3 Projection calibration during rehearsal; 3.3.1.4 Testing repertoire extremes in response to acoustics; 3.3.1.5 Rehearsing key sections to save stamina
3.3.2 Principles and hierarchy within acoustical adaptation	3.3.2.1 Musical interpretation and acoustical adaptation; 3.3.2.2 Instrumental adaptation and acoustical adaptation; 3.3.2.3 Prioritising familiarity and proven strategies under performance pressure
3.3.3 Adapting musical elements to ensure communication	3.3.3.1 Tempo, articulation, breathing, and pedal adjustments for clarity; 3.3.3.2 Clarity and voicing; 3.3.3.3 Dynamics and projection
3.3.4 Instrumental and spatial adjustments	3.3.4.1 Adjustments to piano lid position; 3.3.4.2 Adjustments to instrument placement; 3.3.4.3 Adjustments to acoustical properties
3.3.5 Reflective listening, playback, and audience recalibration	3.3.5.1 Self-playing piano and reflective listening; 3.3.5.2 Recording and playback as review tools; 3.3.5.3 External listening as recalibration of judgement; 3.3.5.4 Simulated and physical acoustics in performance experience

**Table 5:** Domain 4. Learning and developing acoustical awareness: themes and sub-themes.

Theme	Sub-themes
3.4.1 Conservatoire training and the development of acoustical adaptation	3.4.1.1 Tacit form of education; 3.4.1.2 Teacher-led but implicit acoustical guidance; 3.4.1.3 Lack of systematic training in acoustical perception and adaptation; 3.4.1.4 Focusing on technique more than listening; 3.4.1.5 Lack of institutional support for students' own exploration of technology-enhanced learning
3.4.2 External references, comparison, and the growth of acoustical judgement	3.4.2.1 Learning through room comparison; 3.4.2.2 External references of acoustical prediction; 3.4.2.3 Limitations of relying on external references
3.4.3 Development of predictive judgement and self-monitoring	3.4.3.1 Aural training and categorisation of venues based on acoustic predictability; 3.4.3.2 Learning through mediated replay and reflective comparison; 3.4.3.3 From rehearsal to performance as developmental process
3.4.4 Technology and self-evaluation tools	3.4.4.1 Recording as reflective learning route; 3.4.4.2 SPIRIO | r and self-playing playback as learning support; 3.4.4.3 Tools supporting audience-area listening in training; 3.4.4.4 Technology as reflective support, not a replacement for live acoustical experience
3.4.5 Adaptation as a learnable skill	3.4.5.1 From tacit reaction to more stable self-monitoring; 3.4.5.2 Adaptation as closer to technique than abstract theory; 3.4.5.3 Years of exposure and accumulated judgement; 3.4.5.4 Acoustical adaptation remains non-transferable across instruments and halls

#### 3.3.1 Theme: pre-performance evaluation, rehearsal, and listening adjustment

Before performance, rehearsal gave pianists a limited opportunity to learn how the room, piano, and repertoire interacted. Participants used this time to listen to reflected sound, test the instrument's response, and identify material most likely to become unclear, over-resonant, or under-projected. S10 described attention to how the room reflected sound and how the instrument behaved in that room (SM1-S10-Q1), while S4 treated a run-through as a way of gaining a general idea of the adaptation needed (SM1-S4-Q1). This early orientation led to more targeted checks of reverberation, projection, repertoire extremes, and key sections. Because rehearsal time and stamina were limited, pianists selected the sounds and passages most likely to require acoustical adjustment.

##### 3.3.1.1 Evaluating reverberation through impulse sounds

Some performers evaluated reverberation through short sounds, chords, or impulse-like tests. Such tests made decay more audible before the performer committed to a full passage. The speed of the decay helped them judge how long sound remained active in the room and whether later material might become blurred (SM1-S1-Q6).

##### 3.3.1.2 Rehearsal for identifying a general idea of the adaptation

Rehearsal often reduced uncertainty rather than removing it. The pianist may gather enough information to choose a direction without knowing every audience-side consequence. Rehearsal therefore functions as orientation and risk reduction, not as full prediction.

“if it's like a run through before a concert, you can have a general idea.” (S4, SM1-S4-Q1)

##### 3.3.1.3 Projection calibration during rehearsal

Projection calibration involved checking whether sound carried beyond the keyboard position. Pianists tested this against a receiving point in the room, such as a teacher, listener, or jury position (SM1-S4-Q2). As in Section 3.1.1.5, Projection of quiet sound, projection depended on whether sound remained audible and shaped at distance. Recordings provided another external reference for later listening, as discussed in Section 3.3.5.2, Recording and playback as review tools.

##### 3.3.1.4 Testing repertoire extremes in response to acoustics

Testing repertoire extremes was a pragmatic way to use limited rehearsal time. Participants focused on material most likely to expose acoustical problems: loud passages with pedal, thick textures, wide keyboard ranges, and very soft or dry single-line writing. If these extremes worked, the performer had a stronger basis for judging the rest of the programme.

“I'll probably try like the extremes of my program, like the fortissimo bits with full pedal [...] and then the bits which are smallest and driest.” (S2, SM1-S2-Q9)

##### 3.3.1.5 Rehearsing key sections to save stamina

Rehearsing key sections also protected stamina. Pianists could not always spend long periods testing the hall without exhausting themselves before performance. Selective rehearsal balanced physical conservation with acoustical checking. This strategy responds to the constraint described in Section 3.2.2.3, insufficient rehearsal time at performance venue: when time and energy are limited, the performer has to choose which passages most urgently require acoustical testing (SM1-S2-Q10).

#### 3.3.2 Theme: principles and hierarchy within acoustical adaptation

Pianists prioritised adaptation rather than changing everything equally. They tried to protect the musical interpretation while adjusting what could be changed safely: touch, balance, pedal, articulation, tempo within limits, and projection. The hierarchy explains why some changes are safer than others: certain adjustments preserve the identity of the performance, while others risk changing the interpretative structure itself.

##### 3.3.2.1 Musical interpretation and acoustical adaptation

Musical interpretation remained the reference point for adaptation. S6 treated acoustical adjustment as finding a version of the interpretation that suited the room's conditions, rather than abandoning the interpretation itself (SM1-S6-Q3). Acoustical adaptation was artistic as well as technical: the performer changed means in order to preserve musical purpose.

##### 3.3.2.2 Instrumental adaptation and acoustical adaptation

Instrumental adaptation and acoustical adaptation were closely linked. Participants often had to distinguish between the piano's action, pitch behaviour, pedal response, tonal limits, and the room's reflected sound. This distinction is especially important for solo pianists because the venue generally supplies the piano while other musicians supply their own, familiar instrument.

“So we're not just listening to the instrument [...] we're adapting our physical approach to play, according to what we hear.” (P1, SM1-P1-Q6)

##### 3.3.2.3 Prioritising familiarity and proven strategies under performance pressure

Under performance pressure, performers tended to prioritise familiar and proven strategies. S10 described reliance on known approaches when attention was already loaded (SM1-S10-Q2). A known way of reducing pedal, clarifying articulation, or testing projection could be safer than a new solution discovered too late.

#### 3.3.3 Theme: adapting musical elements to ensure communication

Musical adaptation appeared most concretely in tempo, articulation, breathing, pedal, voicing, balance, dynamics, and projection. Pianists adjusted these parameters in order to keep musical communication intact. The adjustments were not made in isolation; they formed a connected response to the acoustic conditions.

##### 3.3.3.1 Tempo, articulation, breathing, and pedal adjustments for clarity

Tempo, articulation, breathing, and pedal shaped whether musical detail remained clear in resonant or dry spaces. Participants treated tempo as a fundamental parameter, but one that could be difficult to change once an interpretation had been formed:

“the one parameter that is most fundamental and also the hardest to change is tempo.” (S2, SM1-S2-Q11)

Greater reverberation could also affect articulation. S11 explained that a passage previously played staccato might need to become “even more detached and shorter” in a hall with more reverberation:

“in a hall with more reverberation I would often make it even more detached and shorter.” (S11, SM1-S11-Q1)

Articulation and note length could therefore become ways of managing how long sounds remained connected in the room. Pedal and pacing were tied to the same clarity problem. Too much pedal or rushed movement could make notes, harmonies, or textures overlap, so that musical lines became less distinct. S4 used “wash” to describe this loss of separation:

“not rushing too much and not using too much pedal. Otherwise, everything just becomes a wash.” (S4, SM1-S4-Q3)

##### 3.3.3.2 Clarity and voicing

Clarity and voicing required the pianist to decide which material should remain present in the hall. This involved more than playing louder. Participants described pitch-specific listening, hand balance, and musical-element balance as ways of making the intended structure audible. In Phase 2, S6 connected pitch response directly to balance decisions, so voicing appeared as a response to how the instrument and room redistributed musical material.

“I will try to understand how every register of the instrument reacts to the acoustics. And I would base the balance between the hands or the balance between certain musical elements.” (S6, SM1-S6-Q4)

##### 3.3.3.3 Dynamics and projection

Dynamics and projection were adjusted according to both room support and communicative need. A supportive hall could allow softer sound to travel, while a dry or absorptive space could require more active projection. Dynamic marking and effective projection are not identical. The performer may need to modify dynamic effort so that the musical relationship, not merely the written level, reaches the listener.

“And I'll try to match them so that they're clearly [...] relates to the listener in the most optimal way I can.” (S6, SM1-S6-Q5)

#### 3.3.4 Theme: instrumental and spatial adjustments

Instrumental and spatial adjustment concerned the few strategies that alter the physical or spatial conditions around the performance rather than the interpretation alone. These include how the piano lid is positioned (e.g., fully open, partially open, or closed), the location of the piano on the stage, and adjustment of acoustic-modifying features of the space such as panels or curtains.

##### 3.3.4.1 Adjustments to piano lid position

Where available, lid position offered a practical way to modify projection and balance before changing the interpretation itself. It could affect how much sound entered the room and how directly it reached listeners. Because this adjustment changes the physical setup rather than the interpretation, it may reduce the need for more extreme changes to touch, tempo, dynamics, or pedal in Section 3.3.3, Adapting musical elements to ensure communication (SM1-S11-Q2).

##### 3.3.4.2 Adjustments to instrument placement

Instrument placement could affect how sound entered the room and how the pianist heard the response. The strategy depended heavily on venue flexibility and was not always available. Its inclusion expands adaptation beyond the player's body: the instrument's position in the room can shape the performer's own hearing and the sound heard from the audience position (SM1-S1-Q7).

##### 3.3.4.3 Adjustments to acoustical properties

Some venues offer the possibility of adjusting the acoustical properties of the space through physical or electronic means. Participants noted, however, that this was often not possible because of the features of the performance space or because they did not have adequate permission or support to make such adjustments. When the environment could not be modified, the performer had to compensate through playing. Physical adjustment was therefore useful where possible, but musical adjustment remained necessary when the room itself could not be changed.

“so we had to adjust ourselves to the acoustics rather than adjusting the acoustics to ourselves.” (S1, SM1-S1-Q8)

#### 3.3.5 Theme: reflective listening, playback, and audience recalibration

Reflective listening addresses a practical limitation of keyboard-side hearing: the pianist cannot normally hear from the audience while playing. Playback, self-playing piano, and external listeners become ways of recalibrating stage-side judgement. These tools do not replace live experience. Their value lies in allowing performers to compare the perspective they occupy while playing with perspectives they cannot occupy at the same time.

##### 3.3.5.1 Self-playing pianos and reflective listening

The use of a self-playing piano such as the Steinway SPIRIO | r used in Phase 2 made it possible to hear one's own performance without simultaneously producing it. Separating listening from bodily execution supported more reflective judgement and allowed the performer to experience their performance as an audience member might. In S7′s Phase 2 reflection, the value of this separation lay in gaining a perspective on how the performance sounded beyond the immediate experience of playing.

“Gives you a more objective perspective on how you're playing.” (S7, SM1-S7-Q5)

##### 3.3.5.2 Recording and playback as review tools

Audio playback allowed performers to compare intended sound, remembered physical effort, and heard result from outside the act of playing. Its acoustical value lay in making listener-position difference more available: microphones placed nearer to or farther from the piano could help performers hear how sound reached different parts of the hall. P1 described distant microphones as a way of accessing a listener-side reference that the pianist could not occupy while playing:

“mics which are further away [...] will give this sense of how it sounds to the person who's paid the 10 p to get the seat at the back of the hall. [...] it's very hard to adjust to what you‘re not listening to [...] if you've got the experiences of hearing how this sounds, through microphones, or playing back recordings, then you will know exactly what's happened” (P1, SM1-P1-Q10)

Recording and playback therefore supported acoustical recalibration by helping performers compare keyboard-side hearing with a recorded listener-side reference. The tool remained a precaution rather than a substitute for live rehearsal or audience-position listening, as developed in Section 3.4.4.4.

##### 3.3.5.3 External listening as recalibration of judgement

External listening helped performers recalibrate bench-side assumptions. Teachers, peers, or hall-side listeners could report what the pianist could not hear while playing. The external listener did not become an authority over interpretation, but they could offer feedback from the position where communication would actually be judged.

“the only way to help that is possibly to sit in the audience yourself in the venue that you're going to play in.” (S2, SM1-S2-Q12)

##### 3.3.5.4 Simulated and physical acoustics in performance experience

The simulated acoustic environment helped reduce unfamiliarity before participants entered the physical hall. It did not remove the need for adaptation, but it offered performers a preparatory reference. S7′s Phase 2 reflection treated venue simulation as a bridge between rehearsal and venue, not as a substitute for live acoustical experience.

“if you go to rehearse to this hall with your professor, and before that you went to the simulator [...] I think you'll perform so much better.” (S7, SM1-S7-Q3)

### 3.4 Domain 4. Learning and developing acoustical awareness

Acoustical awareness is developed through tacit exposure, teacher guidance, room comparison, external references, predictive judgement, playback, technology, and accumulated experience. These findings support acoustical adaptation as a learnable skill, but not as a rulebook that can be transferred unchanged from venue to venue. Pianists require the development of skills leading to a reliable process of listening, testing, comparing, and adjusting rather than a fixed sequence of stages. Domain 4 integrates both phases. Phase 1 established its initial pedagogical and developmental structure, while Phase 2 contributed more of the situated evidence on simulation, playback, audience-position comparison, and reflective self-monitoring.

Table 5 summarises the themes and sub-themes within Domain 4.

#### 3.4.1 Theme: conservatoire training and the development of acoustical adaptation

A key pedagogical problem identified was that acoustical guidance existed but was often unevenly organised and taught. Participants reported receiving guidance about sound, projection, listening, and hall response, but this guidance was often embedded within standard lessons at their instrument rather than organised as explicit and systematic acoustical training. This resulted in the perception of an uneven educational pathway with some students learning through teachers and experience, while others lacked a systematic framework for developing the same skill.

##### 3.4.1.1 Tacit form of education

Learning about acoustics was often embedded in lessons, rehearsals, and performance experience rather than presented as a named or formalised curriculum area. Tacit learning can be powerful because it occurs close to musical action, but it also depends heavily on opportunity, teacher awareness, and the student's ability to notice what is being taught indirectly (SM1-P1-Q7).

##### 3.4.1.2 Teacher-led but implicit acoustical guidance

Teachers played an important role, but their guidance could remain brief or implicit rather than systematic. In Phase 2, S7 recognised the value of teacher feedback from the back of the hall, while other student reflections distinguished such feedback from formal training. These accounts indicate that conservatoire pedagogy already contains acoustical knowledge, but often in a fragmented form.

“my teacher has helped me a little in this area... not really formal training, just some brief pointers.” (S12, SM1-S12-Q1)

##### 3.4.1.3 Lack of systematic training in acoustical perception and adaptation

Participants' comments pointed to a gap between the importance of acoustical adaptation and the lack of formal training devoted to it. The absence of a course did not mean absence of learning, but it did mean that students may have to assemble the skill from scattered experiences rather than from a structured developmental sequence.

“There is no such course.” (S12, SM1-S12-Q2)

##### 3.4.1.4 Focusing on technique more than listening

Participants' accounts suggested that conservatoire training could foreground technique while leaving room-listening less explicitly developed. Technique remained central because it was one of the main routes through which adaptation happened. The pedagogical issue is that technical control and acoustical listening need to be taught together rather than treated as separate areas of musicianship (SM1-S7-Q6).

##### 3.4.1.5 Lack of institutional support for students' own exploration of technology-enhanced learning

Participants saw technology as potentially useful, but access, guidance, and institutional structure affected whether students could use playback, simulation, or audience-position listening as part of systematic learning. Without institutional support, these tools risk remaining occasional novelties rather than integrated routes for reflective practice (SM1-S7-Q6).

#### 3.4.2 Theme: external references, comparison, and the growth of acoustical judgement

Participants' judgement grew through comparison. Pianists built acoustical understanding by encountering different rooms, different pianos, and different listening positions. External references from teachers, peers, and recordings could help, but they did not remove the need for performers to test acoustical information through their own playing. Pedagogy and experience met when outside information was tested in relation to the performer's own sound.

##### 3.4.2.1 Learning through room comparison

Room comparison helped performers build a repertoire of expectations. By moving between dry, resonant, small, large, simulated, and physical environments, pianists could learn which features were consequential for their own playing. The learning was cumulative because no single room could teach the full range of possible responses (SM1-S13-Q1).

##### 3.4.2.2 External references of acoustical prediction

Teachers, peers, venue staff, and outside listeners could provide predictive references before or during rehearsal. These references helped performers anticipate what they might need to test. They were most useful when they guided attention towards likely problem areas without replacing the performer's own listening (SM1-S2-Q13).

##### 3.4.2.3 Limitations of relying on external references

External references remained limited because another person's report could not fully replace the performer's own experience of touch, instrument response, and stage hearing. Recordings also had limitations because microphone placement and playback conditions changed what was heard. The performer still had to convert outside information into usable action at the keyboard (SM1-S1-Q12).

#### 3.4.3 Theme: development of predictive judgement and self-monitoring

Predictive judgement appeared when performers could anticipate acoustical difference before it became disruptive. Prediction did not mean certainty. It meant learning to recognise likely vulnerabilities before they became serious problems: blurred articulation, weak projection, unstable balance, or excessive resonance. Self-monitoring linked this anticipation to action during rehearsal and performance.

##### 3.4.3.1 Aural training and categorisation of venues based on acoustic predictability

P1′s account framed prediction as a skill developed through aural training and informal categorisation (SM1-P1-Q2). Performers could learn to recognise types of rooms and estimate which musical parameters were likely to become vulnerable. Such categorisation remained flexible because the piano, repertoire, and audience condition could change the result.

##### 3.4.3.2 Learning through mediated replay and reflective comparison

Replay and playback supported reflective comparison by letting performers hear the same performance from another position or after the event. They made otherwise unavailable differences easier to notice. The tool revealed how the performance changed when separated from the act of producing it; its value was not limited to exposing mistakes (SM1-S7-Q7).

##### 3.4.3.3 From rehearsal to performance as developmental process

The transition from rehearsal to performance was itself developmental. Each transition taught performers where preparation held, where it failed, and how acoustical decisions had to be made under pressure. S2′s account of hoping that rehearsal work was enough shows why this developmental process remains uncertain: the performer often learns only afterwards whether rehearsal decisions were adequate.

“whatever I've done in the rehearsal time was enough.” (S2, SM1-S2-Q14)

#### 3.4.4 Theme: technology and self-evaluation tools

Recording, playback, self-playing piano, simulation work, and audience-area listening supported acoustical learning by placing the performer outside their usual keyboard-side hearing position. They made it possible to compare the intended performance with another version of the same event. The pedagogical caution is clear in the participant accounts: technology can support reflective listening, but it cannot replace live room experience.

##### 3.4.4.1 Recording as reflective learning route

Recording worked as an external ear. It helped performers compare what they thought they produced with what was captured, even though recording conditions shaped what could be heard. Recordings are therefore useful for reflection but cannot be treated as transparent copies of hall experience (SM1-S7-Q8).

##### 3.4.4.2 SPIRIO | r and self-playing playback as learning support

The SPIRIO | r system extended this reflective route by allowing participants to hear their own performances from outside the act of playing and how they interacted with the room's acoustics. In S7′s Phase 2 reflection, the value of this separation lay in listening without the immediate burden of touch, memory, or execution.

“then I can sort of... forget about everything and just hear it.” (S7, SM1-S7-Q9)

##### 3.4.4.3 Tools supporting audience-area listening in training

Audience-area listening tools concern technologies or listening procedures that can help pianists hear their playing from beyond the keyboard position. These tools may support a listener-position perspective through external listener feedback or, as in this study, replay through a self-playing instrument. Their shared purpose is to give pianists access to audience-area listening that is normally unavailable while they perform, supporting comparison between what they feel and intend at the keyboard and what may be heard away from the instrument. S2′s response suggested that this pedagogical use was not always obvious to performers, even when the relevant technology or listening procedure was available:

“I can't believe I've not thought of that [...] how that it can actually be used for this purpose.” (S2, SM1-S2-Q15)

##### 3.4.4.4 Technology as reflective support, not a replacement for live acoustical experience

Participants also identified the limits of technological realism. S6 described hearing the self-playing piano as technically faithful but missing the bodily feel of playing. Technology can relocate listening, but live acoustical experience still includes touch, presence, risk, and the performer's physical relation with the instrument.

“What I played, but I'm not there. And I prefer to hear a less realistic recording where I am actually there.” (S6, SM1-S6-Q6)

#### 3.4.5 Theme: adaptation as a learnable skill

Adaptation becomes learnable when performers improve at noticing cues, testing rooms, comparing perspectives, and selecting effective adjustments. It is not fully transferable because each hall, piano, repertoire, and performance condition changes the task. The skill lies less in memorising solutions than in developing a reliable adaptive process.

##### 3.4.5.1 From tacit reaction to more stable self-monitoring

Development could involve a shift from immediate reaction towards more stable self-monitoring. Performers became better able to notice what the room was doing and to connect that noticing with specific musical choices. Self-monitoring does not remove uncertainty, but it reduces the gap between sensing a problem and selecting a response (SM1-S5-Q6).

##### 3.4.5.2 Adaptation as closer to technique than abstract theory

Participants framed adaptation as a practical skill closer to technique than to abstract acoustical theory. The claim does not reject conceptual knowledge; it locates that knowledge inside action. Acoustical concepts become useful when they help the pianist decide how to touch, pedal, balance, time, or project in a real space.

“I think it is closer to technique.” (S12, SM1-S12-Q3)

##### 3.4.5.3 Years of exposure and accumulated judgement

No single room or instrument provided a complete model for later performance. S7 described this long-term development through repeated encounters with different halls and pianos. Experience expanded participants' set of comparisons and increased confidence in testing, rather than providing a fixed solution that could simply be carried from one venue to another.

“It's really about years and years and years of practice of being a pianist and coming [...] to a different hall and to a different piano.” (S7, SM1-S7-Q10)

##### 3.4.5.4 Acoustical adaptation remains non-transferable across instruments and halls

Acoustical adaptation remained non-transferable in a simple rule-based sense. What travelled was not a fixed solution, but a more reliable process for listening, testing, comparing, and adjusting. The same pianist might need different responses when the hall, piano, repertoire, audience, and psychological context changed. P1 described this through the difference between an empty and occupied hall:

“The difference, as I said between the empty hall and the full hall can be devastating […] ones really got to get the experience of what to do and to be able to adapt very quickly” (P1, SM1-P1-Q9)

This recalls Section 3.2.4.4, Acoustical maladaptation due to fixated thinking, where P1 warned against carrying an “ideal” way of playing or an “ideal acoustic” from one hall or piano to another (SM1-P1-Q8). In this sub-theme, the emphasis shifts to accumulated experience: repeated exposure can make the pianist quicker at recognising change, but it does not turn adaptation into a portable solution.

## 4 Discussion

### 4.1 Principal findings

This study presents solo pianists' acoustical adaptation as a practical, performer-centred form of musicianship. Participants did not treat acoustics mainly as a set of technical descriptors or objective measurement. They understood room response through musical consequences: reflected sound and touch at the keyboard; pitch balance, timbre, and individual-voice clarity; and the relation between projection, stage-audience discrepancy, and feedback from listeners or playback. Adaptation was shaped by conditions already present before performance, including practice-room habits, limited rehearsal access, venue-provided instruments, inherited room conditions, and psychological pressure.

The Phase 2 scenarios extended the interview findings by showing how acoustical judgements operated during situated performance conditions rather than only in retrospective explanation. Quiet projection and dry practice-room habits showed how preparation could carry acoustic assumptions into performance, while simulation-to-hall preparation, rapid adjustment, and playback-based listening showed how those assumptions could be tested or revised. Across the dataset, pianists worked through musical parameters such as tempo, pedal, articulation, voicing, dynamics, projection, and pitch balance, alongside spatial or instrumental adjustment where these were available. Technology may support comparison and recalibration, but participants did not treat it as a replacement for live rehearsal or physical venue experience.

**Figure 1:**
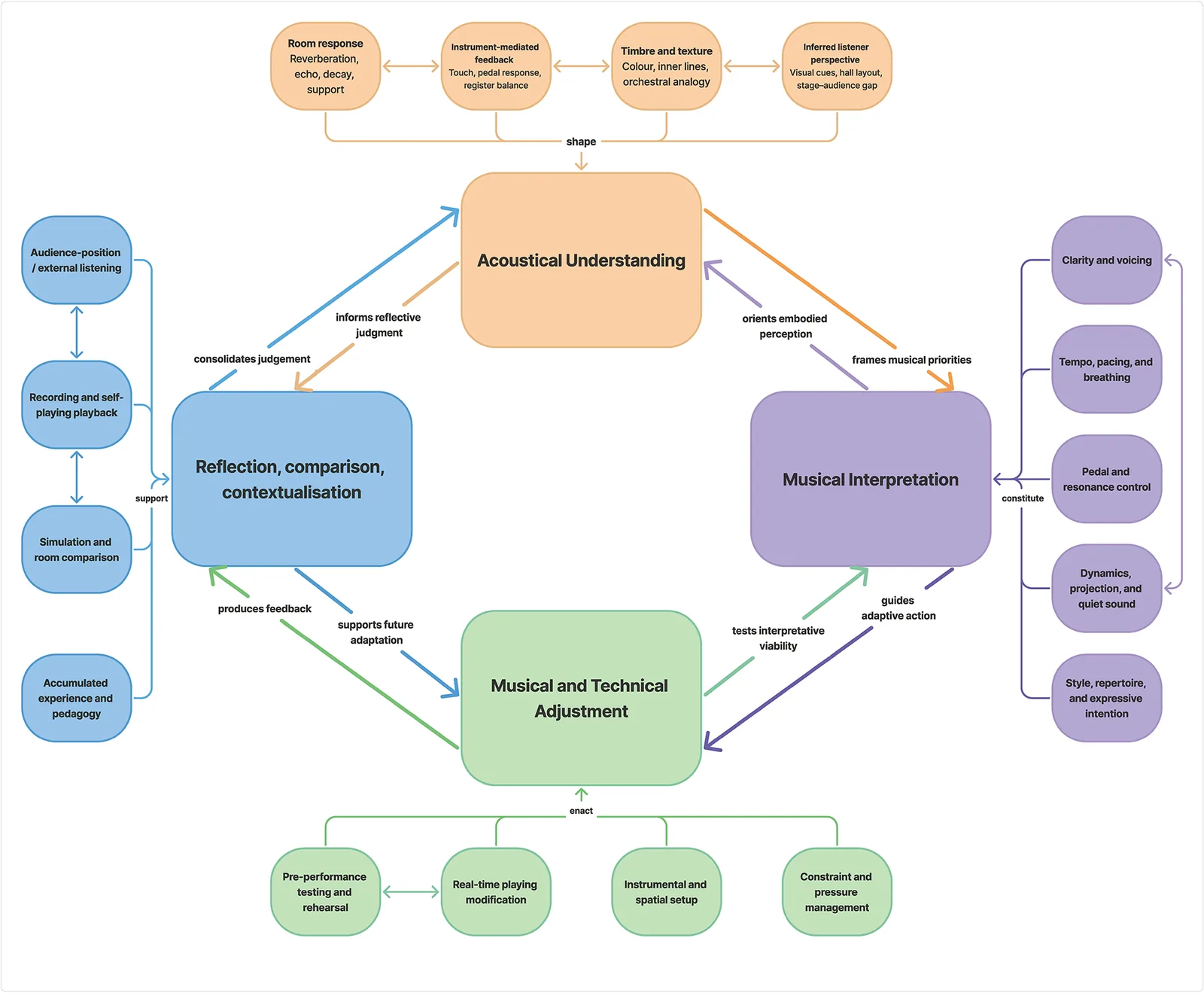
Network model of solo pianists' acoustical adaptation. The model represents acoustical adaptation as a reciprocal process involving acoustical understanding, musical interpretation, performative adjustment, and reflection, comparison, and contextualisation. The surrounding elements represent the acoustical, instrumental, musical, and contextual factors that inform these domains. Paired directional arrows indicate reciprocal but functionally distinct relationships rather than a fixed sequential cycle.

Figure 1 summarises these relations as a reciprocal adaptation network rather than a linear sequence. The four central domains influence one another through functionally distinct pathways, with feedback and reflection enabling performers to revise acoustical judgement over time. Existing performer-side acoustics research has modelled musician perception as an interaction between room response, performance action, and sensory feedback, and has shown that room effects may appear in performance features rather than in verbal judgement alone (Schärer Kalkandjiev, 2015; Schärer Kalkandjiev and Weinzierl, 2013, p. 1–2; Ueno et al., 2010). The present model adds pianist-specific detail: the room is encountered through the venue piano and through pitch-wide, polyphonic textures, while the player's incomplete access to audience-side sound makes comparison, feedback, and repeated exposure central to learning.

### 4.2 Performer-side acoustical understanding, tacit knowledge, and listener-position discrepancy

Concert-hall and music-acoustics literature provides established terms for room response, but those terms do not by themselves explain how pianists use acoustical information while playing. Beranek and Meyer relate musical experience to hall and instrumental conditions, and Hyde revisits intimacy and initial-time-delay gap (Beranek, 1992, 2004; Hyde, 2019; Meyer, 2009). The present data show how such information becomes practical at the keyboard: pianists judged acoustics through touch and pitch response, especially where these affected voicing clarity, projection, timbre, and the audibility of individual voices.

Polanyi's (1966) account of tacit knowledge helps clarify why this knowledge was often easier for participants to enact than to verbalise. Pianists do not usually attend to the hand, key resistance, pedal response, and returned sound as separate objects. They use these cues while aiming at a musical result in the hall. In that sense, the instrument becomes the immediate route through which the performer works towards a more distant sounding outcome. This does not make performer language imprecise or deficient. It means that much acoustic knowledge is embedded in skilled action before it becomes available as explicit explanation.

(Schön 1983) distinction between reflection-in-action and reflection-on-action further clarifies how acoustical adaptation develops over time. Participants demonstrated reflection-in-action when they monitored decay, clarity, balance, touch, and projection while playing and adjusted their actions according to the sounding result. Reflection-on-action occurred through playback, post-scenario interviews, and audience-position listening, allowing performers to examine aspects of performance that were unavailable from the keyboard position. Importantly, these processes were not separate stages: retrospective comparison created new perceptual references that could inform later real-time adjustment. Acoustical adaptation therefore developed through an ongoing relationship between embodied action, external feedback, and accumulated judgement.

This tacit structure also helps explain why stage-audience discrepancy mattered so strongly. Stage-acoustics studies distinguish musician priorities from audience-side listening, especially around support, reverberance, clarity, and mutual audibility (Gade, 1989a,b; Marshall et al., 1978; Ueno and Tachibana, 2003, 2005, 2011; Wenmaekers, 2017). Listener-side studies also show that listening position and programme material, as well as distance and individual taste, can shape hall judgement (Kuusinen and Lokki, 2015; Lokki and Pätynen, 2020; Lokki et al., 2016). In this study, that position difference became a rehearsal and learning problem. Teachers sitting at the back of the hall, SPIRIO playback, recordings, and audience-position listening helped participants compare keyboard-side sensation with sound heard elsewhere.

Performer terminology therefore needs careful handling. (Schärer Kalkandjiev and Weinzierl 2013, p. 2) note that performers use diverse terminology and that their primary response to acoustics may appear in playing rather than verbal report. In the present study, terms such as dry, wet, clear, echoey, bright, supportive, or blurred organised action. They helped pianists decide what to alter in playing or preparation, including pedal, tempo, voicing, touch, projection, and rehearsal priority. Sachs's interviews with pianists support this emphasis on listening and interpretative judgement, although they are not room-acoustics research (Sachs, 2009). The contribution here is to show how performer language, bodily feedback, and listener-position uncertainty work together inside acoustical adaptation.

### 4.3 Adaptation under constraint: room, instrument, and repertoire

Empirical work has shown that performance behaviour can change under different acoustic conditions. Bolzinger et al. (1994) studied professional pianists using a Disklavier in controlled variable acoustics; Ueno et al. (2010) developed a performer-centred model; Kato et al. (2015) extended this work through audio analysis; and Berg et al. (2016) and Jullander et al. (2019)) connect room acoustics with interpretation. The present data help explain why such changes may be musically necessary. Pianists altered or considered altering tempo, pedal, voicing, articulation, dynamics, and projection because the room changed whether the intended musical structure remained communicable.

An ecological perspective helps sharpen this point without turning the findings into a general theory of environment. Room acoustics can be understood as part of the action environment for performance: they provide opportunities for as well as barriers to certain musical outcomes (Clarke, 1988, 2005). A reverberant hall may constrain clarity in rapid or dense textures while affording sustain, warmth, or timbral blend. A dry room may expose articulation and support detail while making legato, soft projection, or resonance harder to sustain. Participants' accounts carried this double character: acoustics could support or hinder performance by changing the practical conditions under which a musical intention could be realised.

For pianists, this environmental relation is intensified by the venue instrument. The player often adapts to an unfamiliar room and an unfamiliar piano at the same time. Touch, action, pedal response, tonal balance, pitch behaviour, and room support are difficult to separate in use because the pianist encounters the room through the instrument. This makes the piano case distinctive within performer-side acoustics research. A hall may be preferred, but the analytical issue is whether a given instrument-room relation allows the performer to keep control of balance, polyphonic clarity, tone colour, and projection.

Practice rooms formed part of the same constraint system. Research on rehearsal and practice rooms argues that these spaces need acoustical attention in their own right (Hatlevik, 2012; Ryherd, 2008; Wenmaekers, 2017). In the present data, daily practice environments shaped effort, dynamic scale, pedal use, and projection before participants entered the performance venue. This matters pedagogically because habits formed in dry, small, noisy, or otherwise restricted rooms may not transfer smoothly to larger or more reverberant spaces.

These strategies should not be read as universal prescriptions. Clarity, projection, resonance, and communication are not stable ideals across all repertoire. Polyphonic writing may make individual-voice clarity especially salient; dense Romantic textures may require a different balance of resonance and bass support; and Impressionist or contemporary repertoire may sometimes value blur, atmosphere, or sustained sonority rather than maximum separation. The findings therefore identify strategy categories for timing, pedal, voicing, articulation, projection, pitch balance, and listening, rather than style-neutral rules. Piano performance literature on pedalling, tonal imagination, and pitch colour supports this repertoire-sensitive reading (Banowetz, 1985; Neuhaus, 1973).

### 4.4 Technology, feedback, and reflective learning

Technology was most useful when it made otherwise inaccessible relations available for comparison. Playback, self-playing piano, and audience-position listening helped participants hear a version of the performance separated from the act of producing it. Schön (1983) concept of reflection-on-action is useful here: the performer can examine the result after, or outside, the immediate bodily task of playing. In this study, that separation helped participants compare intention, bodily memory, and heard result.

Research on adjustable and virtual acoustics provides a useful comparison because altered room response can support experiment or training when the setup remains perceptually credible and musically useful (Barron, 1978; Carlsson, 2015; Chen, 2022; Freiheit, 2003, 2004, 2010; Geerdes, 1975; Pätynen, 2007; Vigliensoni et al., 2017). Risset and Van Duyne's computer-controlled acoustic piano is also a useful precedent, although the present study used self-playing playback for reflection rather than real-time interaction (Risset and Van Duyne, 1996).

Recording and playback literature clarifies the same issue from another direction. (Volioti and Williamon 2017, p. 499–500) describe recordings as resources for preparation and self-regulated learning, with students relying on them especially strongly. Such technologies, particularly through their ability to support the process of reviewing practice to ensure that planned goals are met, can support a reflective cycle of self-regulated learning (Waddell and Williamon, 2024). The present playback tasks extend this logic to acoustical adaptation. Participants used playback to compare bench-side intention with heard result, while also recognising that recorded or simulated listening cannot reproduce touch, audience presence, physical risk, or the full pressure of live performance.

Simulation should therefore be understood as a differently structured learning environment that enables performers to compare acoustic perspectives that are otherwise difficult to access, while remaining distinct from live performance conditions. It can support preparation by making contrast, perspective, and feedback more available, while live acoustical experience still includes bodily production, room-instrument interaction, and performance pressure. The value of technology lies in supporting comparison and reflection, and it aligns with technology-enhanced music learning research (Ramirez-Melendez and Waddell, 2022) addressing musicians' demand for learning technologies that can fit established practice routines (Waddell and Williamon, 2019).

### 4.5 Psychological pressure and adaptive expertise

The findings suggest that acoustical uncertainty is a specific psychological pressure within performance preparation. Participants described anxiety around wet acoustics, unpredictable rooms, competitive contexts, and uncertainty about whether adjustments were effective. Performance anxiety research has already shown that pressure can narrow attention and disturb flexible monitoring (Kenny, 2011; Lehmann et al., 2007; Williamon, 2004, p. 233–235). The present data add that acoustical uncertainty may be one route through which this pressure enters performance decision-making.

The distinction between routine and adaptive expertise is useful here if used cautiously. (Hatano and Inagaki 1986) distinction between routine and adaptive expertise helps explain why acoustical adaptation requires more than transferring previously successful solutions. Pianists could rely on familiar strategies under pressure, but changing relationships among room, instrument, repertoire, and listening position meant that these strategies required continual reassessment. A familiar way of reducing pedal, clarifying articulation, or testing projection may be safer than a new solution found too late. Adaptive expertise in this context involved recognising when an established response remained appropriate and when the acoustic situation required modification.

This balance between routine and flexibility is one reason participants emphasised the value of the rehearsal within the space where performance would take place. Rehearsal did not remove uncertainty entirely, but it allowed participants to identify vulnerable passages, test extremes, compare perspectives, and reduce the attentional burden of making every acoustical decision during performance itself. Adaptive skill, in this context, is not endless flexibility. It is the capacity to prepare enough reference points that real-time adjustment becomes more manageable.

### 4.6 Pedagogical implications

The findings support a cautious case for more explicit acoustical awareness training in conservatoire education. They do not justify a universal method. Students may benefit from clearer procedures for comparing rooms and testing vulnerable passages, then using audience-position listening to translate feedback into musical action. (Williamon and Thompson 2006, p. 411–412, 425) argue that applied research should inform conservatoire training and show that students may turn first to instrumental teachers for performance-related problems, work increasingly implemented in conservatoire settings (e.g., Waddell et al., 2025b). For acoustical adaptation, teacher advice may become more useful when linked to repeatable listening and testing procedures.

A possible pedagogical approach could combine structured comparison between dry, wet, and hall-like spaces with guided audience-position listening, simulation-based preparation, and reflective playback tasks that separate playing from listening. Teachers could encourage students to distinguish between perceived acoustic information, inferred consequences, and intended adjustments, thereby making the implicit judgement process underlying acoustical adaptation more explicit. This would connect acoustical awareness with the metacognitive self-monitoring associated with self-regulated learning (McPherson and Zimmerman, 2002, 2011), while still keeping the work close to playing.

These suggestions are directions for development, not tested interventions. The data support more explicit training, but do not prove which teaching format is most effective. A systematic pedagogy would need to respect repertoire, instrument, venue, and performer differences and avoid turning adaptation into a checklist. The aim is to help students build a more reliable process of listening, testing, comparing, and adjusting.

### 4.7 Limitations and future research

The study has several limits in scope. The sample was small and situated within one institutional context, the Royal College of Music, with access to purpose-built simulation and playback facilities that are not typical of most conservatoires. The findings should therefore be read as a detailed qualitative account rather than as a population-level claim. In qualitative terms, the contribution lies in transferability and conceptual insight rather than statistical generalisation. Future research could compare students and professionals across institutions, including conservatoires without comparable simulation or playback infrastructure, to examine how acoustical adaptation develops under different educational and practical conditions.

The design covered broad domains: acoustical understanding, real-world constraints, adaptation strategies, technology, and learning. This supported a connected model of acoustical adaptation, but limited the depth given to each domain. The study was designed to examine shared processes of acoustical adaptation rather than differences between demographic groups, training stages, or repertoire categories. The professor-student composition of Phase 1 is relevant: P1 contributed a distinct expertise-level voice that was important for several themes; readers should interpret those themes as combining student perspectives with a strong expert account, rather than as evenly distributed across all participants. Future research could investigate how these factors shape the development and expression of adaptive strategies.

Phase 2 was not designed as a controlled experiment isolating instrument, lid position, repertoire, or room effects. The completed records support protocol-level interpretation across acoustical contrast, simulation, playback, and reflection, but they do not support controlled claims about specific instrument manipulations or participant-by-participant differences in repertoire demand. Findings about room-instrument interaction should therefore be read as contextual and interpretive. Future studies could link qualitative performer accounts with objective acoustical measurements and performance data, so that pianist-described categories such as support, dryness, projection, and blurriness can be examined alongside measurable room features and recorded performance changes. Score use and memorisation were not standardised, so the study cannot determine whether playing from memory facilitated musical communication or acoustical adaptation.

Observation conditions also could not fully reproduce the pressure of public performance, examination, or competition. Simulation and playback provided valuable comparison, but participants' accounts indicated that these tools do not fully reproduce audience presence, emotional pressure, physical risk, or every aspect of room-instrument interaction. Future studies could examine how simulation systems and self-playing piano systems can be integrated into conservatoire pedagogy while remaining clear about their limits as substitutes for live performance.

The study focused on solo pianists. Some findings may resonate with other instrumentalists, but the piano case has specific features: venue-provided instruments, pitch-wide texture, pedal complexity, and polyphonic clarity. Further research could examine other instruments and ensemble settings to determine which aspects of acoustical adaptation are instrument-specific and which reflect broader performer-side acoustical learning. Longitudinal designs following students through structured acoustical training and into public performance would provide stronger evidence for whether these approaches improve adaptive judgement over time.

## 5 Conclusion

The findings identify acoustical adaptation as part of how solo pianists prepare, judge, and communicate performance. Solo pianists understood acoustics through musical consequences: reflected sound and touch at the keyboard; clarity of individual voices and wider voicing; projection and timbre; and stage-audience hearing differences. Their acoustic knowledge was often tacit, because it was enacted through the instrument before it could be fully articulated as theory. Adaptation was also constrained. Practice rooms, rehearsal access, venue-provided instruments, psychological pressure, and incomplete audience-side information shaped what performers could judge and change.

Pianists adapted through connected musical and technical decisions involving timing, pedalling, articulation, voicing, dynamics, projection, and pitch balance. Participants associated this skill with repeated experience, comparison, feedback, and reflection. Technology may support this development when it helps performers compare perspectives and separate playing from listening, but it should not be treated as a replacement for live acoustical experience. The study contributes a qualitative model in which perception, constraint, strategy, feedback, and learning remain closely connected. It positions acoustical adaptation as a learnable form of practical musicianship shaped by the room, the instrument, the body, and the listener's position.

## Data Availability

The original contributions presented in the study are included in the article/Supplementary material, further inquiries can be directed to the corresponding author.
