# Supplementary material for: Playing to the room: how solo pianists perceive, understand, and adapt to acoustic conditions
Source: Front Psychol. 2026 Aug 12;17:1909431. doi: 10.3389/fpsyg.2026.1909431 (PMC13506470; doi:10.3389/fpsyg.2026.1909431)
Supplement: Supplementary file 1 [file Supplementary_File_1.docx]

# 1 Supplementary Material 1 - Participant quotation register

This supplementary material lists the participant quotations cited in the article. Quote IDs are used in the main text and in this register to link each cited excerpt to its fuller source context. The table provides the full quotation or full translated source text used for the article, and original-language wording where relevant. The quote-ID format is functional: for example, SM1-S1-Q1 refers to Quote 1 by student participant S1 in Supplementary Material 1. In this format, SM1 identifies Supplementary Material 1, S1 identifies the anonymised participant, and Q1 identifies that participant's first cited quotation in this register.

| **Quote ID** | **Source quotation / full English translation** | **Original-language wording, where applicable** |
| --- | --- | --- |
| SM1-S1-Q1 | so I'm just trying to hear what's going on in the room if there is enough echo, if there is enough reverberation |  |
| SM1-S2-Q1 | We can't touch the acoustics cause it's in the air [...] Um, and you need to be very, very comfortable with your hands and the instrument before you can start trying to adjust to acoustics […] |  |
| SM1-P1-Q1 | how many people practice the alto and tenor voices together in a fugue? […] it is the one thing which is important, people practice soprano, familiar places, (but) how many people actually know what the alto voice sounds [like] against the bass voice […] the tenor voice against the soprano? […] Not only just will that show how well we know the fugue, but it also shows how those voices interrelate. |  |
| SM1-P1-Q2 | But measuring, listening to measure the decay of sounds at different levels, different areas of the keyboard is very good aural training, and (we need) lots of that. |  |
| SM1-S1-Q2 | if I play something very quiet would it carry out to the end of the hall things like that |  |
| SM1-S5-Q1 | It's like to have a sound center… it's a bit like what you were saying about if you played two songs, but it's like an actor that can project even like whispering in a big hall that you will still hear it. |  |
| SM1-S3-Q1 | Sound is really, really hard to quantify... especially in music... because everyone expresses things differently... so it is hard to quantify what the numbers should be. | 声音就挺难挺难定量的…尤其是音乐嘛…因为是每个人表达不一样…就有点难用量化去表达数字应该怎样 |
| SM1-S5-Q3 | As [the other participant] said, like you can only do so much sitting at the piano. Your perception of it will be very different to the hall. |  |
| SM1-S6-Q1 | And the acoustics and the space and the audience and all of that is extremely important to me, of course. But it is, again, a reactionary instinctive process. |  |
| SM1-P1-Q3 | The more we think like an orchestra the better […] If we were conducting, and we said well, we need those muted horns in the middle to be more resonant and be more colorful… we need more violas. And we need to balance the lower woodwind more consciously […] We're thinking about different parts of our hands, we're thinking about different touch different ways from our body to do that […] In other words, you are in a sense, an orchestra. |  |
| SM1-P1-Q4 | of course it is an imitation, just as legato is the imitation […] that the human voice can be legato but piano really can't […] But we have the capacity to evoke a legato, if we are very good craftspeople with our sound and our balance. |  |
| SM1-S6-Q2 | Certainly, I will try to understand how every register of the instrument reacts to the acoustics. And I would base the balance between the hands or the balance between certain musical elements. |  |
| SM1-S8-Q13 | But in terms of visual effect... or that kind of feeling... I think the real thing would probably bring pressure more quickly. | 但是我觉得视觉效果上……或者是那种感觉上来……就是我觉得真正……可能带给我压力会更快。 |
| SM1-S3-Q2 | A concert hall may have many people, and the decorations above may also act like sound-receiving or reflecting panels; tone color reflection and wall surfaces are variable factors. | 就像是音乐厅也会有很多人，可能那些上面的装饰啊，也是收音版啊。音色要怎样反射啊 […] 可能就会浪费了本来就很好的钢琴 […] 欸墙也是一个variable thing |
| SM1-S7-Q1 | Usually, usually all the teachers I had, usually in the rehearsals and in the concert halls, they would sit closer towards the back and then they would report how it sounds from there. |  |
| SM1-S7-Q2 | And that's, yeah, I think that's how I learned. So you learn, you learn how it sounds here and then how it translates into the back of the hall. |  |
| SM1-S2-Q2 | Exactly, yeah, because what you hear and what they hear can be very, very different. |  |
| SM1-S1-Q3 | …if you keep practicing in a [student-accommodation] practice room, your muscles will get used to playing at a certain volume… so let's say you go to a bigger concert hall which is very dry, which means you will have to use more force… to produce more sound. Then you actually are changing your interpretation because you weren't playing that hard before. |  |
| SM1-S5-Q7 | those pods are very drying [...] my environment is different than hers. |  |
| SM1-S2-Q3 | because yeah all of the rooms in college you can hear the traffic outside… that really just hurts the immersion. |  |
| SM1-S12-Q18 | The lighting can be useful. | 灯光是可以用的。 |
| SM1-S9-Q1 | obviously if you're less familiar with a place, you're having to try and adapt on the go. |  |
| SM1-S1-Q4 | …acoustics are not under your control… but what is under your control is your response to the acoustics. So let's say you happen to be in a very dry room, therefore it's not really reflecting to the end of the hall, you can do something about it in your playing, rather than change the acoustics of the room. You can't really do that. |  |
| SM1-P1-Q5 | Okay, that's what they've got to do. But first of all, to get to know the acoustics of the place you're in, according to the piano that you're playing… it is not just the acoustic, it's the piano too. This is not your own instrument. It's another instrument. We have to get the experience of that. |  |
| SM1-S1-Q5 | …because we didn't have any rehearsal in the concert hall before the performance day, we didn't know how it was gonna sound at all. So I actually asked one of my teachers to come into the concert hall to listen to us, so that we could decide on the staging altogether. So the end result… was the final solution for the acoustics. |  |
| SM1-S2-Q4 | because sometimes you might be in a place where you're on stage and you think… it's all fine and then later you find out that the bass was not enough or the top was not enough… But to you on stage it sounded enough… there's just… no definite way of finding out by yourself how it sounds from the audience. |  |
| SM1-S5-Q2 | With the softer passages, again, it's projection, isn't it? So in like a concert hall, you can play softer then in that acoustic because you know it will carry. Whereas in a practice room, you have to project. |  |
| SM1-S1-Q10 | I think to hear each other is more important than to see each other |  |
| SM1-S1-Q11 | if what you are hearing is together then it is not really necessary to see each other all the time |  |
| SM1-S5-Q4 | I find in some halls it's... some halls are less forgiving of you not having a center. |  |
| SM1-S2-Q5 | because there have been instances, I think much, much early on when I was an undergrad, where I used to have a problem with underplaying, like for example, not playing full out. And part of the reason why that happens sometimes was that I would be worried about the clarity and stuff. So I'll start to play small, you know, as a sort of like a very unproductive response to that worry. |  |
| SM1-S5-Q5 | The second one, I could sing because you can hear and you can connect the sounds between the two. If it was in the first acoustic, I would tend to practice more technique in that acoustic... because it's dull. |  |
| SM1-P1-Q8 | you can't take that around from piano to piano, from hall to hall… you have to be able to adapt… …we can't simply think… I played this way, this is my ideal way, this is my ideal acoustic… and you can't transport that you have to be able to adapt. And that is going to be a matter of profound listening. |  |
| SM1-S7-Q4 | Then it gives me some head space to focus on other things like acoustics, communication… |  |
| SM1-S2-Q7 | There's only been one time where the acoustic heavily affected my interpretation and it was that [concert hall] where we played concerto… because it was extremely swimmy… that was the main anxiety point for everybody… I think that's been the only time in my career where I made, like, a huge, huge adjustment. I played many sections of my Bach Toccata… slower. |  |
| SM1-S8-Q1 | If I am in a place where I cannot clearly hear my own playing, or where the whole reverberation is very heavy and affects how I receive the sound, I become anxious. | 但是如果我在一个……我自己甚至不能很清楚地听见我的演奏的东西，或者是它整个混响非常重……它会影响到我对那个声音的接收的时候……我就会很焦虑。 |
| SM1-S2-Q8 | Yeah. And then you sort of worry, oh my goodness, if I'm not adjusting to this, then what if somebody is adjusting to this better than I'm adjusting to it? But, you know, you could call that part of the competition anxiety in which acoustic is only one element of so many other elements. |  |
| SM1-S9-Q2 | But I think I was able to react quite quickly. Whether or not my adjustments were effective is a different matter. |  |
| SM1-S10-Q1 | When I actually go on stage and perform, I do not think of it first; before going on stage or backstage, I can basically work out how I will adapt to that state. | 但我真正上台、真正演的时候，我不会第一考虑……我在上台前、在后台，我基本上也能处理好我接下来要怎么去适应这个状态。 |
| SM1-S4-Q1 | To answer your point, I would say, yeah, you if it's like a run through before a concert, you can have a general idea. |  |
| SM1-S1-Q6 | so for example I clap or I go play something on the piano and try to hear how it sounds to me first of all… and you get an immediate feeling of the room… oh wow it's very dry or it's very boomy… and then you try to adjust yourself instinctively this is not something you logically think about you just try different versions… and if possible again you ask somebody to listen to you from several places in the… hall… so what parameters I'm looking for is literally instinctive reaction to what's going on …not visual at all it's only audio |  |
| SM1-S4-Q2 | Whereas in the [concert hall], I was playing to [listener] and this other guy. So I had to actively project to them… |  |
| SM1-S2-Q9 | then I'll probably try like the extremes of my program, like the fortissimo bits with full pedal, bigger keyboard range, thicker textures, and then the bits which are smallest and driest [...] To try out the extremes, basically. |  |
| SM1-S2-Q10 | we don't have time to run through the whole piece. You, there's no stamina for that. It's not, it's not a good use of energy. So go for like a few spots, which you know will be important or potentially problematic… the places where you think have the highest potential to become messy… there's no fixed… every piece of music you play, once you become familiar with it, you start to recognize the problematics… And then when you go and rehearse, you just target those spots specifically. And not play the whole piece… There isn't enough time. There isn't enough energy. |  |
| SM1-S6-Q3 | When I go on stage, I try to create a bridge between what the composer meant and wanted to say and how I feel at the moment. And this is my top priority. |  |
| SM1-P1-Q6 | So we're not just listening to the instrument, or not just methodically doing something that we practice, we're adapting our physical approach to play, according to what we hear. That is the craft. |  |
| SM1-S10-Q2 | If halfway through playing I suddenly feel that the acoustic effect does not match what I expected, I would not really change it again, because that would somewhat break my own emotional viewpoint. | 如果我在弹到一半的时候……突然感觉到……跟我期待的这个音响效果不是很符合，但我不太会再去变了，因为那样的话会让我觉得……会有点打破我自己一个情绪的观点。 |
| SM1-S2-Q11 | Yeah, I think […] it makes more sense, at least for me, to adjust […] to build in extra time […] the one parameter that is most fundamental and also the hardest to change is tempo. Everything else, you can kind of change on the spot [...] You can alter, like, the other parameters [...] like, you know, pedal, voicing. |  |
| SM1-S11-Q1 | In some halls with a lot of reverberation, your articulation sometimes changes... for example, I may have played this place staccato before, but in a hall with more reverberation I would often make it even more detached and shorter. | 在一些混响大的厅，你的 articulation 有时候会变……比如说我这个地方之前只是弹 staccato，但是呢，我可能在回响大厅里的时候常常会更跳、更短。 |
| SM1-S4-Q3 | And in a combination with not rushing too much and not using too much pedal. Otherwise, everything just becomes a wash and you don't really hear the notes properly. |  |
| SM1-S6-Q4 | Certainly, I will try to understand how every register of the instrument reacts to the acoustics. And I would base the balance between the hands or the balance between certain musical elements. |  |
| SM1-S6-Q5 | And I'll try to match them so that they're clearly… how should I say? Clearly relates to the listener in the most optimal way I can. |  |
| SM1-S11-Q2 | Researcher: So, in Classical repertoire, does changing the acoustics make a bigger difference than opening or closing the lid? Participant: It is definitely greater than that. | Researcher: 所以声学在古典的时候改变比开盖关盖更大吗？ Participant: 肯定要大于它。 |
| SM1-S1-Q7 | …before the performance day we didn't know how it was gonna sound at all, so we actually—I actually asked my teacher, one of my teachers, to come into the concert hall to listen to us, so that we could decide on the staging altogether. So the end result… was the final solution for the acoustics… we actually changed the placement of the players until we came up with the final solution. |  |
| SM1-S1-Q8 | We do but we didn't use them… I don't think we had them available to us at that very moment. I didn't see them around. They're usually at the side of the hall… so we had to adjust ourselves to the acoustics rather than adjusting the acoustics to ourselves. |  |
| SM1-S7-Q5 | Gives you a more objective perspective on how you're playing. |  |
| SM1-P1-Q10 | We can of course, record… we can listen back, providing the reproduction is of very high quality. Well, you have, as you say, the opportunity of recording, close mics, and mics which are further away. And those further away will give this sense of how it sounds to the person who's paid the 10 p to get the seat at the back of the hall. it's very hard to adjust to what you're not listening to, because as a performer, you're hearing something very different sitting up on the stage, from those people that at the back of the concert hall. But if you've got the experiences of hearing how this sounds, through microphones, or playing back recordings, then you will know exactly what's happened… But you still can't second guess it, you can just simply take precautions. |  |
| SM1-S2-Q12 | Um, as for like being trained, to be honest, I'm not sure if we've been trained to listen to acoustics because, um, your professor can tell you to listen, but then you're listening to what you hear at the piano, which is very different from, uh, what the audience hears, which I think has been a recurring theme today is me saying, "Oh, I have no idea what they hear over there". Um, and the only way to help that is possibly to sit in the audience yourself in the venue that you're going to play in. |  |
| SM1-S7-Q3 | Like, if you go to rehearse to this hall with your professor, and before that you went to the simulator and you simulated with different acoustics, I think you'll perform so much better. |  |
| SM1-P1-Q7 | Well, I think we do, we don't do it as a specific science that way. I mean, if you take a Bach Fugue in four parts, we practice the parts separately, very often, the touch for each part is going to be different, the dynamic controls do not always coincide. Because the polyphony is changing. And therefore, we're adapting to each part in a different way… in terms of touch, maybe non-legato, maybe legato with a little pedal, or do we pedal the beginning of the note; midway through the note… all sorts of finite things. |  |
| SM1-S12-Q1 | But my teacher has helped me a little in this area... not really formal training, just some brief pointers. | 但是我的导师是有帮我做过这方面的……一个简单的……不算培训吧，就是简单的提点。 |
| SM1-S12-Q2 | There is no such course. | 没有这份课程。 |
| SM1-S7-Q6 | When I was doing it, the technology wasn't as reliable. So like recording yourself with the phone, for example, just didn't reflect the reality. |  |
| SM1-S13-Q1 | The same piano, in different acoustics, I think your judgment of the piano will be different. | 同样的琴……在不一样的声学里面……我觉得你对这个琴的判断会不一样。 |
| SM1-S2-Q13 | …you could have a friend or a professor and then they can tell you oh it sounds like this like this and then you can only imagine based on what they just described to you… and then the next best thing is to get somebody to play and then you go to the audience. But then it's different because of the play… |  |
| SM1-S1-Q12 | So what I mean by trust is not really personal goals or aims but trust in hearing… to be first of all efficient, and the same in the same artistic values… for example, the reason why I thought the basses was too little [was] because it wasn't good balance and I wanted more sort of grounded sound. But let's say there was a person who wanted thinner sound and more crispy—maybe for them the basses should be less. |  |
| SM1-S7-Q7 | It diminishes the gap between, as I said, between you and the listener. |  |
| SM1-S2-Q14 | I'll try to adjust whatever I can during the rehearsal time. And then when it came to the actual performance, I'll just not think about that and hope that whatever I've done in the rehearsal time was enough. |  |
| SM1-S7-Q8 | There's a reason why I'm heavily dependent on technology. |  |
| SM1-S7-Q9 | Because, well, then I can sort of… forget about everything and just hear it. |  |
| SM1-S2-Q15 | yeah I’ve seen real Spirios and they really do sound like someone's playing them on the spot… actually I can't believe I’ve not thought of that… like but I’ve not thought about how that it can actually be used for this purpose |  |
| SM1-S6-Q6 | What I played, but I'm not there. And I prefer to hear a less realistic recording where I am actually there. |  |
| SM1-S5-Q6 | If it was in the first acoustic, I would tend to practice more technique in that acoustic… because it's dull. |  |
| SM1-S12-Q3 | I think it is closer to technique. | 我觉得是偏向于技术。 |
| SM1-S7-Q10 | It's really about years and years and years of practice of being a pianist and coming, you know, to a different hall and to a different piano. |  |
| SM1-P1-Q9 | The difference, as I said between the empty hall and the full hall can be devastating […] ones really got to get the experience of what to do and to be able to adapt very quickly |  |
