# Supplementary material for: Playing to the room: how solo pianists perceive, understand, and adapt to acoustic conditions
Source: Front Psychol. 2026 Aug 12;17:1909431. doi: 10.3389/fpsyg.2026.1909431 (PMC13506470; doi:10.3389/fpsyg.2026.1909431)
Supplement: Supplementary file 2 [file Supplementary_File_2.docx]

# 1 Supplementary Material 2 – Interview Schedule and Observation Procedure

This supplementary material provides the indicative Phase 1 interview schedule and the Phase 2 observation procedure. It also lists the setup details that should be documented when recreating or extending the observation scenarios.

## 1.1 Phase 1 indicative interview schedule

The Phase 1 interviews were semi-structured. The following schedule indicates the main interview areas and the prompts used to guide follow-up questioning.

| **Focus area** | **Questions and probes** |
| --- | --- |
| Starter question | - How do you think different acoustics may affect your practice and performances? |
| Acoustical adaptation strategies | - What would you do to adapt to the acoustics? - How do you practice this process of acoustic adaptation? - When you do not have access to a specific performance venue, how would you prepare to adapt to its acoustics? - Is there anything that could help you prepare for different acoustical environments? |
| Technology utilisation | - Are you using technology to help you with your acoustic adaptation? - What kind of technology are you using and how effective are they? - Have you used, or have heard of, acoustical simulation or auralisation technologies? - How would you feel about using such systems? - What kind of technology do you imagine could be useful for adapting to the acoustics? - Would you consider using these technologies? Why? |
| Pedagogy | - Have you been taught to listen to and prepare for the acoustics? If so, how was that being taught? - Do you teach your students to listen to and prepare for the acoustics? If so, how? |

## 1.2 Phase 2 observation procedure

Participants could take part in up to four observation scenarios and could choose to take part in as many or as few as they wished. Each observation scenario lasted approximately 15–30 minutes. Before and after each observation, participants took part in a reflective interview of approximately 5–15 minutes. A complete scenario session, including interviews, therefore lasted approximately 1 to 2 hours.

Before each scenario, participants discussed their first impression of the acoustic environment, their sense of the acoustics, and any potential strategies for adapting to the acoustics. After each scenario, they took part in a short reflective interview about their experience and thoughts regarding the acoustics.

Participants did not need to prepare anything before the observation. They could be asked to practice and/or perform one piece or a combination of pieces they were currently working on, and they could also be asked to sight-read. The scenarios and interviews were audio-video recorded and transcribed for analysis, subject to participant consent.

### 1.2.1 Scenario 1: Practising in acoustically contrasting spaces

For this scenario, participants were invited to practice at the piano for approximately 10–15 minutes, following their own practice routine. The piano lid was off for the practice-room conditions. The first practice condition used a low-reverberation preset to mimic a dry practice-room acoustic. Participants then practised comparable or related material in a contrasting preset with a moderate amount of reverberation. The Meyer Sound Constellation system was operated through available presets rather than by reporting exact reverberation-time values; therefore, the procedure is described in terms of relative acoustic contrast rather than precise RT measurements.

**Operating order:**

1. Researcher explained the scenario, asked short pre-scenario questions about first impression, perceived acoustics, and possible strategy.
2. Participants practised selected material in the dry/low-reverberation condition, with the piano lid off.
3. Researcher altered the acoustic character of the room by switching presets on the Meyer Sound Constellation system.
4. Participants practised comparable or related material in the contrasting condition.
5. Researcher documented comments, testing behaviours, and visible playing changes.
6. Researcher conducted the post-scenario interview.

### 1.2.2 Scenario 2: Spontaneous adaptation to acoustical changes

Participants performed selected material in a simulated acoustic environment. During playing, the acoustic condition was changed without explicitly notifying the participant. The piano remained the same; the changing condition was the acoustic simulation. Changes were created by switching the Meyer Sound Constellation system on or off and/or by switching between available acoustic presets. Exact reverberation-time values were not collected for this scenario, so the scenario is reported as a relative contrast between preset conditions.

**Operating order:**

1. Researcher explained the scenario, asked short pre-scenario questions about first impression and expected strategy.
2. Participants began playing in the initial simulated acoustic condition.
3. Researcher changed the simulated acoustic condition during playing by switching the Meyer Sound Constellation system on or off and/or switching between available acoustic presets.
4. Participants continued playing unless they naturally stopped or commented.
5. Researcher documented verbal reactions and apparent changes in playing.
6. Researcher conducted the post-scenario interview.

### 1.2.3 Scenario 3: Self-reflection from the audience’s perspective

Participants recorded their playing using the Steinway SPIRIO | r system. The piano then reproduced the performance while participants listened from different positions within the room, first from the piano-stool position and then from one or more audience-side positions.

**Operating order:**

1. Participants recorded into the SPIRIO | r playback system.
2. Researcher explained the scenario, then started piano playback.
3. Participants listened from the piano position.
4. Participants moved to one or more audience-side positions and compared the listening positions.
5. Researcher documented the participants’ reactions and self-reflection.
6. Researcher conducted the post-scenario interview.

### 1.2.4 Scenario 4: From virtual rehearsal environment to actual performance space

Participants first worked in a virtual rehearsal environment that mimicked a physical performance space, then moved to the actual performance space on which the virtual setting was based and played the same or related material. The comparison was between the simulated preparation condition and the physical performance condition.

**Operating order:**

1. Researcher explained the scenario, asked short pre-scenario questions in or before the virtual rehearsal condition.
2. Participants rehearsed and played selected material in the virtual rehearsal environment.
3. Researcher and participants moved to the physical performance space.
4. Participants rehearsed and played the same or related material.
5. Researcher documented comments, testing behaviour, and comparison between expectation and actual room response.
6. Researcher conducted the post-scenario interview.
